# Protonation state of the S-O-NH crosslink between lysine and cysteine

**DOI:** 10.1039/d6ra07624f

**Published:** 2026-09-22

**Authors:** Danajana V. Wijerathne, Michaela A. Morris, James W. Gauld

**Affiliations:** a Department of Chemistry and Biochemistry, University of Windsor Windsor Ontario N9B 3P4 Canada; b Department of Chemistry, Memorial University of Newfoundland St. John's NL A1C 5S7 Canada jgauld@mun.ca

## Abstract

A new putative oxygen-bridged crosslink between lysine and cysteine was experimentally identified in a transaldolase from the Gram-negative bacterium *Neisseria gonorrhoeae* (NgTAL). This crosslink was proposed to function as a reversible redox switch. Although it has generally been represented in its neutral S-O-NH form, its protonation state remains uncertain. Importantly, protonation may influence both the physicochemical properties of the crosslink and the structure of the surrounding protein environment. In the present study, we used a multiscale computational approach combining QM-cluster, QM/MM, and molecular dynamics simulations to investigate the preferred protonation state of the Cys38-S-O-NH-Lys8 crosslink and the structural consequences of nitrogen protonation. QM-cluster calculations identified the nitrogen centre as having the highest proton affinity (PA) and gas-phase basicity (GPB) across all dielectric environments considered. When the crystallographic water molecules were included explicitly, proton transfer through the water network yielded the N-protonated S-O-NH_2_^+^ species, while no stable S- or O-protonated crosslink was obtained. The QM/MM calculations also favoured nitrogen protonation, giving PA and GPB values of 1700.2 and 1685.5 kJ mol^−1^, respectively. Furthermore, the optimized QM/MM structure revealed an extended hydrogen-bonding network connecting the protonated crosslink to Glu93 through the water molecules. Three independent 200 ns MD simulations further showed that the protonated crosslink maintains a persistent association with Glu93, through a direct hydrogen bond (∼69% occupancy) or a one-water bridge (∼18%), whereas the neutral crosslink most often lacked an interaction with Glu93 (∼66%). Nitrogen protonation also shifted the preferred Glu93 side-chain conformation towards a rotameric state resembling the crystallographic structure and modestly altered the global conformational dynamics of the protein. Collectively, these results consistently support assignment of the NgTAL crosslink as predominantly N-protonated, S-O-NH_2_^+^. Its stabilization arises from the combined influence of the local water network, Glu93, and the surrounding protein environment.

## Abstract 2

The recently identified Cys38-S-O-NH-Lys8 crosslink in *Neisseria gonorrhoeae* transaldolase (NgTAL) exists predominantly in its N-protonated S-O-NH_2_^+^ state.

## Graphical abstract

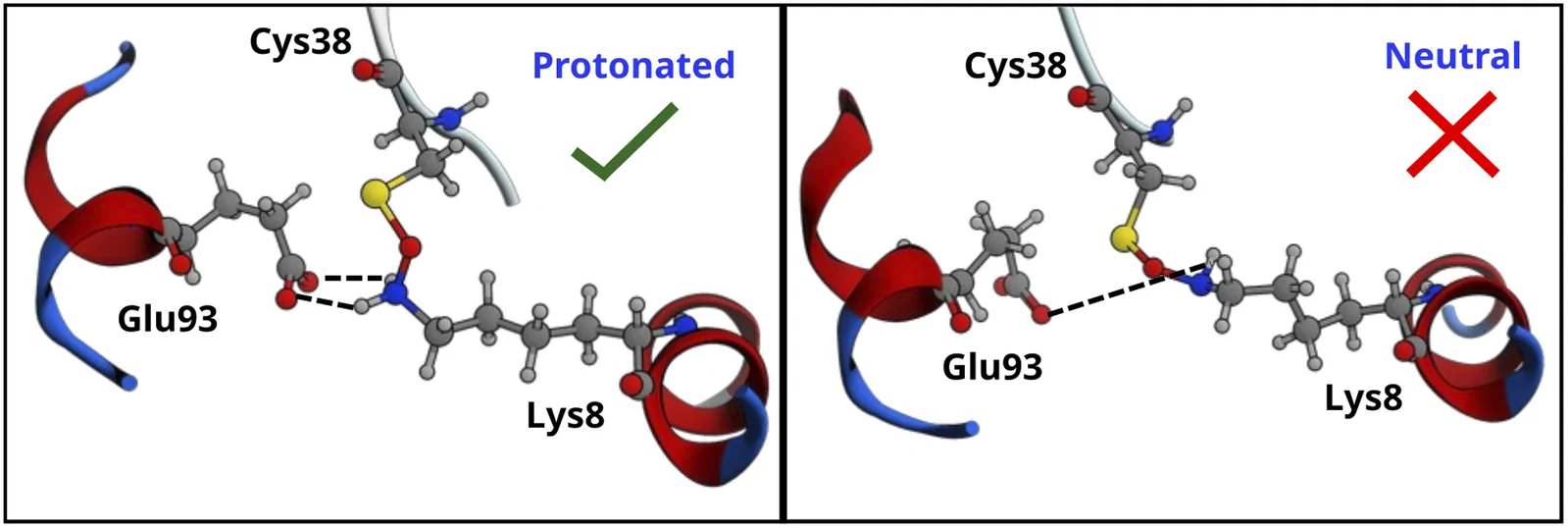

## 1. Introduction

Recently, a remarkable and entirely new putative crosslink was experimentally identified (Fig. 1) in a transaldolase from the Gram-negative bacterium *Neisseria gonorrhoeae* (NgTAL).^1^ This enzyme is involved in the pentose phosphate pathway which is critical for NADPH and ribose biosynthesis. Notably, it was suggested that its functioning could be regulated by the reversible non-metal redox formation of an oxygen bridged covalent crosslink between the sidechains of a cysteine and lysine: that is, the formation of (Cys)S-O-N(Lys) crosslink.^2^ Shortly thereafter it was concluded that such S-O-NH redox switches between lysine and cysteine are ubiquitous in proteins from all domains of life.^1,2^ Most recently, similar S-O-NH crosslinks have also been identified between the sidechains of cysteine and arginine.^3^ This has led to a reevaluation of our understanding of protein architecture and its implications for biological processes. Several studies^4–6^ have proposed or aimed to elucidate the mechanism(s) for formation of this crosslink. Unfortunately, however, much remains unclear or unknown about this crosslink including, importantly, its exact nature. Indeed, such information is critical to fully understanding the formation and role of this crosslink. Indeed, its possible protonation state(s) and their effects on the structure of the crosslink must properly be established for further studies on its formation mechanism, function, and other related aspects.

**Fig. 1:**
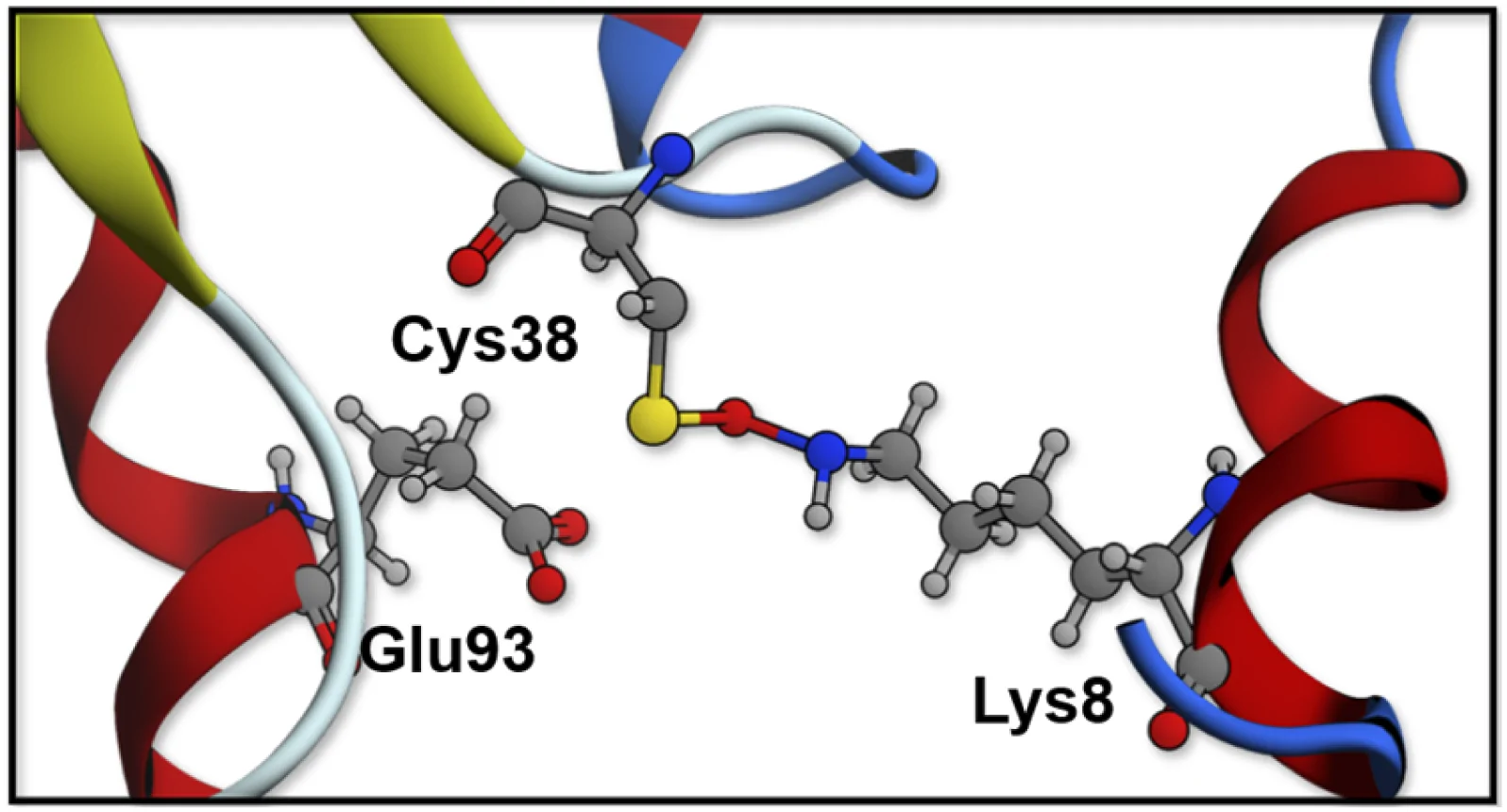
Proposed neutral S-O-NH crosslink between lysyl and cysteinyl residues in transaldolase from the Gram-negative bacterium *Neisseria gonorrhoeae* (NgTAL; PDB ID: 6ZWH).^1^

Previous experimental^1,2,4^ and computational^5^ investigations have generally assumed that this crosslink is neutral; S-O-NH. Protonation is a fundamental and critical biochemical process. For example, it plays vital roles in stabilizing protein structures,^7,8^ metabolic reactions and enzyme catalysis,^9,10^ and functioning of several other biological crosslinks.^11,12^ Importantly, it affects the physicochemical properties of the species being protonated. Thus, for many new biochemical species it is essential to gain an understanding of its protonation-related chemistry. For example, it was discovered that collagen IV, a critical component of the extracellular matrix, contains a biochemically unique interstrand sulfilimine (S=N) crosslink between the sidechain sulfur of a methionyl and the sidechain nitrogen of a lysyl or hydroxylysyl.^11,12^ While it was initially assumed to be neutral, computational studies,^13^ including by our group,^14^ concluded that *in vitro* it is in fact most likely singly protonated. Furthermore, this greatly influenced the S–N bond's length and physicochemical properties, and the distribution of environmental waters around the crosslink.^11^

In this present study, we have used a multi-scale computational approach to investigate the preferred protonation state of the putative S-O-NH crosslink formed between lysine (Lys8) and cysteine (Cys38) in the transaldolase enzyme of NgTAL. More specifically, we have applied a combination of molecular dynamics (MD) simulations, QM-cluster,^15^ and quantum mechanics/molecular mechanics (QM/MM) techniques^16^ to study both various possible protonation states of the S-O-NH crosslink and the resulting effects on the crosslink's structure.

## 2. Computational methods

### 2.1 Small model QM-cluster calculations

The Gaussian 16 software^17^ package was used to perform all QM-cluster calculations. A reduced QM-cluster model was generated from the crystal structure of the S-O-NH crosslinked protein (PDB ID: 7ODP).^2^ The model comprised the crosslinked Lys8 and Cys38 residues together with the three crystallographic water molecules that participate in hydrogen-bonding interactions with the crosslinked moiety. The peptide backbone was truncated at the Cα atoms, and the resulting dangling bonds were saturated with hydrogen atoms. To preserve the crystallographic orientation of the truncated residues, the atoms defining the truncation points were constrained during the initial geometry optimization while allowing the remaining atoms to relax. The resulting optimized structure was subsequently used as the starting model for all proton affinity (PA) and gas-phase basicity (GPB) calculations (Fig. S1A). For comparison, PA and GPB calculations were also performed using an equivalent QM-cluster model without the three crystallographic water molecules (Fig. S1B). All optimized geometries and harmonic vibrational frequencies were obtained at the ωB97XD/6-311G(2d,2p) level of theory.^18^ The corresponding enthalpy corrections (Δ*H*_corr_) and entropies (at 298.15 K) were also obtained at the same level of theory. The surrounding environment (*ε* = 1.0 (gas-phase), *ε* = 4.0 (protein environment), and *ε* = 78.3 (aqueous solution)) was modelled using the default Integral Equation Formalism Polarizable Continuum Model (IEFPCM) method.^19^ To identify an appropriate DFT level of theory for geometry optimization and PA/GPB calculations, an initial benchmark study (Table S1) was carried out using a simplified S-O-NH crosslink model (CH_3_CH_2_-S-O-NH-CH_2_CH_3_). The performance of various DFT methods was evaluated by comparison with reference calculations at the CCSD/6-311G(d,p) level of theory. The PAs and GPBs were calculated using the following equations:^20,21^

1 ^298.15K^PA_A_^−^ = −Δ*H* = −(*H*_AH_ − *H*_A_^−^ − *H*_H_^+^)

2 ^298.15K^GPB_A_^−^ = −Δ*G* = −(−PA − *T*(*S*_AH_ − *S*_A_^−^ − *S*_H_^+^)

### 2.2 QM/MM calculations

The ONIOM QM/MM method,^16^ as implemented in the Gaussian 16 (ref. 17) software package, was used to examine the PAs and GPBs of crosslink atoms (S, O, and N). The electronic embedding (EE) formalism was used for all of the QM/MM optimizations.

The QM/MM models were constructed from the crystal structure (PDB ID: 7ODP)^2^ of the S-O-NH crosslinked NgTAL protein (Fig. 2). To preserve the experimentally determined geometry of the protein and the S-O-NH crosslink, the crystallographic coordinates were maintained throughout model preparation. The crystallographic citrate molecule, a non-native crystallization ligand, was removed, while all crystallographic water molecules were retained. The resulting structure was solvated in an explicit TIP3P water box and neutralized by the addition of counterions.

**Fig. 2:**
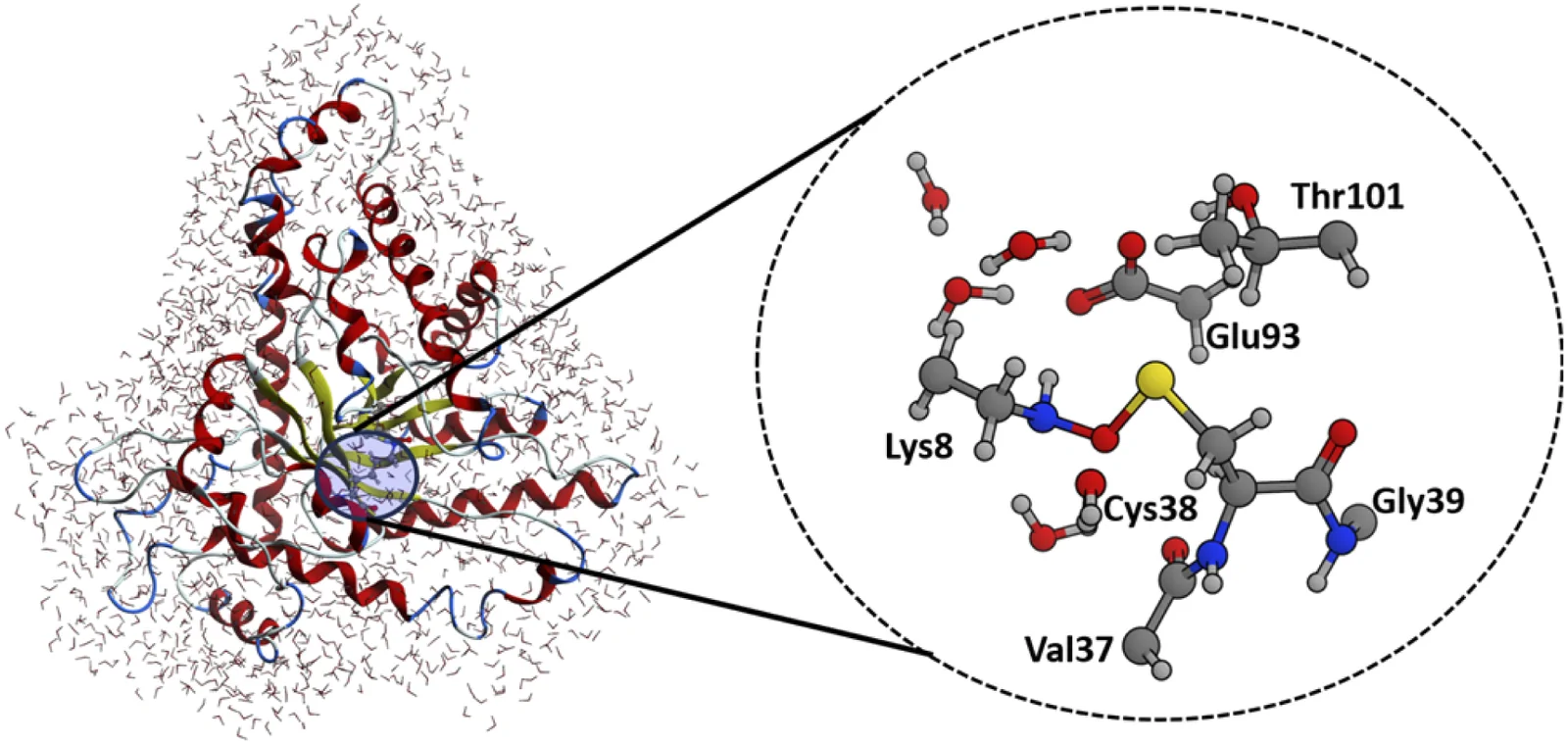
QM/MM model constructed from NgTAL transaldolase (PDB ID: 7ODP) used in this study. For clarity, selected key residues included in the QM layer are highlighted.

Prior to QM/MM calculations, the solvated system was subjected to a short molecular dynamics relaxation protocol. This protocol was designed to relax the solvent environment and eliminate unfavorable contacts while maintaining the experimentally determined crystallographic geometry of the protein and S-O-NH crosslink. It consisted of two stages of energy minimization, including an initial restrained minimization followed by an unrestrained minimization. The system was then gradually heated from 0 to 300 K under constant-volume conditions with positional restraints applied to the protein heavy atoms. This was followed by two successive constant-pressure equilibration stages, the first with positional restraints for 100 ps and the second with gradual release of the restraints over 100 ps. The final equilibrated structure was subsequently used as the starting point for construction of the QM/MM models. Details of the molecular dynamics protocol are provided in the Molecular dynamics methods section.

The QM region (high layer) was described using the ωB97XD/6-311G(2d,2p) level of theory, while the surrounding protein environment (low layer) was modeled using the AMBER force field as implemented in Gaussian 16.^17,22^ That is, optimized geometries and harmonic vibrational frequencies were obtained at the ONIOM(ωB97XD/6-311G(2d,2p):AMBER)-EE level of theory. The corresponding enthalpy corrections (Δ*H*_corr_) and entropies (at 298.15 K) were also obtained at the same level of theory. To calculate the proton affinity and gas-phase basicity, four crosslink models were constructed: neutral (Cys38-S-O-NH-Lys8), nitrogen-protonated (Cys38-S-O-NH_2_^+^-Lys8), oxygen-protonated (Cys38-S-OH^+^-NH-Lys8), and sulfur-protonated (Cys38-SH^+^-O-NH-Lys8).

The high (QM) layer included the crosslinked residues, key active-site residues (Val37, Gly36, Glu93, and Thr101), and nine water molecules (Fig. 2). The PAs and GPBs were calculated using eqn (1) and (2), as described for the QM-cluster models.

### 2.3 Molecular dynamics simulations

The initial structures for the MD simulations were constructed from the crystal structure of the S-O-NH crosslinked NgTAL protein (PDB ID: 7ODP).^2^ To preserve the experimentally determined geometry of the protein and the S-O-NH crosslink, the crystallographic coordinates were maintained throughout model preparation. Namely, the crystallographic citrate molecule, a non-native crystallization ligand, was removed, while all crystallographic water molecules were retained. All the system preparation steps were done using the Molecular Operating Environment (MOE) software.^23^ In total 3 models were constructed: one uncrosslinked (Cys38-SH/Lys8-NH_3_^+^) and 2 cross-linked systems (neutral (Cys38-S-O-NH-Lys8) or protonated (Cys38-S-O-NH_2_^+^-Lys8)).

The protein was described using the AMBER ff14SB force field,^24^ whereas the partial atomic charges for the S-O-NH crosslinked residues were derived using the standard two-stage restrained electrostatic potential (RESP) charge-fitting procedure.^25^ Force-field parameters for the non-standard crosslinked residues were assigned using the General AMBER Force Field (GAFF2).^26^ The resulting force-field parameters for the S-O-NH crosslink were validated by comparison with QM/MM, CCSD, and crystallographic geometries. Details of the parameterization procedure, force-field validation, and representative AMBER input files are provided in the SI (Tables S2–S4).

Each system was solvated in an explicit TIP3P water box with a minimum solute-to-box distance of 10 Å and neutralized by the addition of Na^+^ and Cl^−^ counterions. The solvated systems were subjected to a two-stage energy minimization consisting of restrained and unrestrained minimization, followed by gradual heating from 0 to 300 K under constant-volume (NVT) conditions while applying positional restraints to the protein heavy atoms. Subsequently, the systems were equilibrated under constant-pressure (NPT) conditions in two successive 100 ps stages, with positional restraints maintained during the first stage and gradually released during the second. The resulting equilibrated structures were then used as the starting point for the production simulations.

Production molecular dynamics simulations were performed using the AMBER simulation package^27^ for 200 ns with a 2 fs integration time step under periodic boundary conditions. Covalent bonds involving hydrogen atoms were constrained using the SHAKE algorithm. Temperature was maintained at 300 K using the Langevin thermostat with a collision frequency of 2.0 ps^−1^, while pressure was maintained at 1 atm using isotropic pressure scaling with the Monte Carlo barostat. Long-range electrostatic interactions were treated using the particle mesh Ewald (PME) method,^28^ and a 10 Å cutoff was applied for short-range nonbonded interactions. Three independent MD simulations were performed for each system. Unless otherwise stated, all analyses were carried out using the final 100 ns of each 200 ns production trajectory (Fig. S5). The analysis scripts used for the molecular dynamics trajectory analyses, the AMBER topology and coordinate files for all three simulated systems, and the optimized QM/MM structures reported in this study are publicly available through Zenodo.^29^

#### 2.3.1 Hydrogen-bond network analysis

To investigate the effect of crosslink nitrogen protonation on the local hydrogen-bonding environment, hydrogen-bond interactions between the S-O-NH/S-O-NH_2_^+^ crosslink, Glu93, and surrounding water molecules were analysed over the final 100 ns of each MD simulation. Hydrogen bonds were identified using the MDAnalysis Python library,^30^ employing geometric criteria of a donor–acceptor distance ≤ 3.0 Å and a donor–hydrogen–acceptor angle ≥ 130°. For each trajectory frame, hydrogen-bond networks were classified as (i) direct hydrogen bonds between the crosslink (S-O-NH and S-O-NH_2_^+^) and Glu93, (ii) one-water bridges, (iii) two-water bridges, or (iv) no hydrogen-bonding interaction. The percentage occupancy of each network type and the overall hydrogen-bond network occupancy were calculated for each simulation (Fig. S3). Analyses were performed independently for the three replicate simulations of both the S-O-NH and S-O-NH_2_^+^ systems, and the reported values represent the average across the three replicates.

#### 2.3.2 Crosslink-Glu93 distance analysis

To evaluate the effect of crosslink nitrogen protonation on the interaction with Glu93, the distances between the crosslinked nitrogen atom and the two carboxylate oxygen atoms of Glu93 (N⋯OE1 and N⋯OE2) were analysed over the final 100 ns of each MD simulation using the MDAnalysis Python library. For each trajectory frame, the N⋯OE1 and N⋯OE2 distances were calculated, and the minimum N⋯O distance was determined to account for dynamic switching of the hydrogen-bonding interaction between the two equivalent carboxylate oxygen atoms. Distance analyses were performed independently for the three replicate simulations of both the S-O-NH and S-O-NH_2_^+^ systems. For each replicate, distances were averaged within 1 ns time bins, and the reported time-evolution profiles represent the mean minimum N⋯O distance ± standard deviation calculated across the three independent replicates (Fig. S2).

#### 2.3.3 Glu93 conformational clustering analysis

To characterize the conformational preferences of Glu93 and evaluate the effect of crosslink nitrogen protonation on its side-chain dynamics, conformational clustering was performed using the final 100 ns of each MD simulation. The Glu93 side-chain conformation was described by its *χ*_1_ (N–Cα–Cβ–Cγ) and *χ*_2_ (Cα–Cβ–Cγ–Cδ) dihedral angles, together with the distances between the crosslinked nitrogen atom and the two carboxylate oxygen atoms of Glu93 (N⋯OE1 and N⋯OE2). To account for the periodic nature of the dihedral angles, *χ*_1_ and *χ*_2_ were represented using their sine and cosine components prior to clustering. Frames from the three replicate simulations were combined separately for the S-O-NH and S-O-NH_2_^+^ systems and clustered using the *k*-means algorithm. Clusters were ranked according to their population, with cluster 1 corresponding to the most populated conformational state. Cluster populations were calculated for the combined trajectories and independently for each replicate to assess the reproducibility of the identified conformational states. Representative structures corresponding to the centroid of the most populated cluster were extracted and superimposed onto the experimentally determined crystal structure for structural comparison. Two-dimensional *χ*_1_/*χ*_2_ conformational maps and cluster population distributions were generated for both protonation states, while replicate-specific cluster populations are provided in the SI (Fig. S4).

#### 2.3.4 Root-mean-square deviation (RMSD)

Protein RMSD was calculated to assess the structural stability and conformational dynamics of the S-O-NH, S-O-NH_2_^+^, and uncrosslinked systems. For each of the three independent 200 ns simulations, trajectory frames were first least-squares fitted to the backbone atoms of the corresponding starting structure to remove overall translational and rotational motion. Backbone RMSD values were then calculated relative to this reference structure. Analyses were performed on the final 100 ns of each trajectory and individual RMSD profiles and distributions were reported separately for the three replicate simulations (Fig. S5).

## 3. Results and discussion

Although, as noted above, the crosslink is generally assumed to be neutral, the S-O-NH crosslink has the potential to be protonated at one or more of its three centers: S, O, and/or N. Proton affinities and gas-phase basicities are an effective means of computationally predicting protonation states.^20,21^ Hence, we have considered both the PAs and GPBs of these three crosslink atoms, as well as the effect of the surrounding environment on these properties, using both QM-cluster and QM/MM approaches (Table 1).

**Table 1:** Proton affinities and gas-phase basicities of crosslinked atoms (highlighted in red), calculated at 298.15 K and reported in kJ mol^−1^. Values labeled (W) were obtained using the QM-cluster model including the three crystallographic water molecules, whereas values without (W) were obtained using the corresponding QM-cluster model without these water molecules

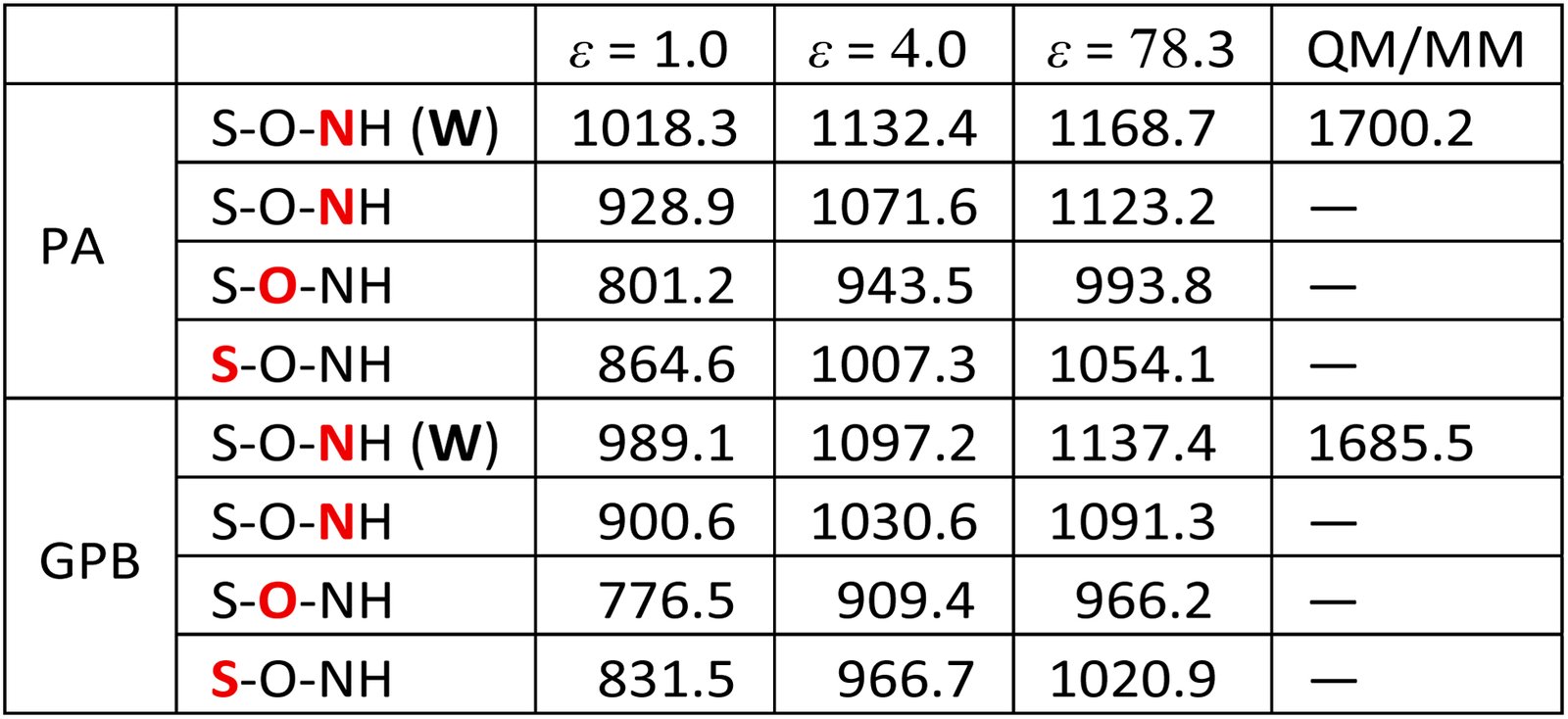

Using the QM-cluster model without the three crystallographic water molecules, the PAs and GPBs of the crosslink S, O, and N centres exhibited the same trend in the gas-phase (*ε* = 1.0), protein (*ε* = 4.0), and aqueous (*ε* = 78.3) environments. Namely, the nitrogen centre had the highest values, followed by the sulfur and then oxygen centres. Furthermore, the calculated values increased as the polarity of the environment increased from *ε* = 1.0 to 4.0 and then 78.3.

As noted, the crosslink nitrogen centre had the highest PA and GPB values at all three dielectric constants considered (*ε* = 1.0, 4.0, and 78.3). More specifically, its PA values were calculated to be 928.9, 1071.6, and 1123.2 kJ mol^−1^, respectively, while the corresponding GPB values were 900.6, 1030.6, and 1091.3 kJ mol^−1^. Thus, increasing the polarity of the environment increased the GPB by 130.0 kJ mol^−1^ on going from *ε* = 1.0 to 4.0 and by a further 60.7 kJ mol^−1^ on increasing *ε* to 78.3. These results indicate that, within the continuum dielectric model, increasing environmental polarity stabilizes the protonated forms of the crosslink. Importantly, however, these calculations do not explicitly account for specific proton-transfer pathways involving protein residues and crystallographic water molecules.

To investigate these effects more explicitly, QM-cluster calculations were subsequently performed with the three crystallographic water molecules included. In contrast to the calculations without the water molecules, the explicit water network provided a pathway for proton transfer to the crosslink nitrogen. Upon structural relaxation, the protons associated with the crystallographic waters were transferred through the water network to the nitrogen centre, yielding the N-protonated S-O-NH_2_^+^ species. No stable S- or O-protonated crosslink species was obtained under these conditions. Within the QM-cluster model, this result provides structural evidence that the local water network can facilitate proton transfer to the crosslink nitrogen and supports the preference for nitrogen protonation predicted by the PA and GPB calculations.

The influence of the explicitly represented protein environment was further examined using QM/MM calculations. These calculations also indicate that protonation at the S and O centres does not lead to stable protonated crosslink structures. In particular, structural relaxation of the initially O-protonated model resulted in transfer of the proton from the crosslink oxygen to nearby Glu93 residue, whereas protonation at the sulfur centre ultimately led to proton transfer toward the crosslink nitrogen. Thus, when the surrounding protein environment and crystallographic waters are explicitly considered, the initially protonated S- and O-centred species undergo proton-transfer rearrangements rather than remaining as stable protonated forms of the crosslink. Notably, the resulting structures consistently favour protonation at the nitrogen centre.

The QM/MM results further support the preference for nitrogen protonation based on the calculated proton affinities and gas-phase basicities. The N-centred crosslink has a PA of 1700.2 kJ mol^−1^ and a GPB of 1685.5 kJ mol^−1^, substantially higher than the corresponding values obtained for the S- and O-centred protonation sites. The notably higher PA and GPB values obtained from the QM/MM calculations, compared with those from the QM-cluster models, likely reflect the influence of the explicitly represented protein environment. In the QM/MM calculations, the quantum mechanical region is treated using electrostatic embedding, allowing the electronic structure of the crosslink to respond to the electrostatic field generated by the surrounding protein. Accordingly, these quantities are best interpreted as thermochemical descriptors within the specified electronically embedded QM/MM model. Their primary significance comes from comparisons among protonation sites treated using the same computational framework.

In addition, the QM/MM model explicitly includes the crystallographic water molecules and the nearby Glu93 residue. This allows the influence of the local hydrogen-bonding network and electrostatic environment on the protonation energetics of the S-O-NH crosslink to be evaluated. As illustrated in Fig. 3A, the protonated crosslink participates in an extended hydrogen-bonding network involving two crystallographic water molecules. These water molecules bridge the protonated nitrogen centre to the carboxylate group of Glu93. The hydrogen bonds exhibit donor–acceptor distances of 2.586, 2.792, 2.656, and 2.960 Å, with corresponding D–H⋯A angles of 172.8°, 151.7°, 163.3°, and 146.6°, respectively. These geometries indicate well-oriented hydrogen-bonding interactions. Collectively, this hydrogen-bonding network delocalizes and stabilizes the positive charge on the N-protonated crosslink. It also provides an indirect hydrogen-bonding pathway that connects the protonated crosslink to the negatively charged Glu93 residue.

**Fig. 3:**
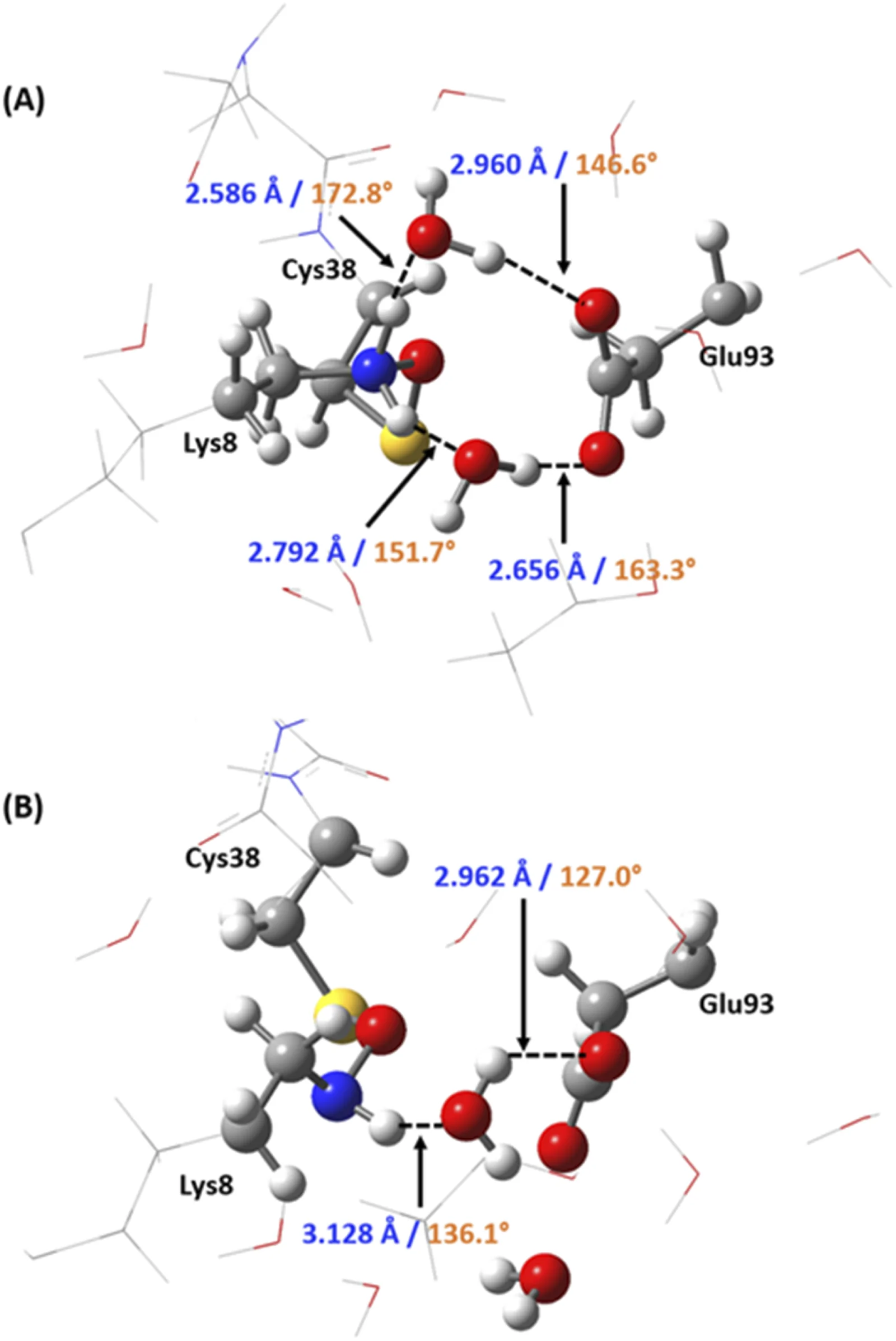
Hydrogen-bonding interactions of the S-O-N crosslink with Glu93 and surrounding water molecules in the optimized QM/MM structures of (A) the protonated S-O-NH_2_^+^ form and (B) the neutral S-O-NH form. Donor–acceptor distances, *r*(D⋯A), and D–H⋯A angles are shown in blue and orange, respectively.

In contrast, the neutral S-O-NH crosslink (Fig. 3B) forms a much less extensive hydrogen-bonding network. Only two hydrogen-bonding interactions are observed, with donor–acceptor distances of 3.128 and 2.962 Å and corresponding D–H⋯A angles of 136.1° and 127.0°, respectively. These longer distances and less favorable hydrogen-bond angles indicate weaker interactions than those observed for the protonated crosslink. Consequently, the neutral crosslink is coupled less effectively to the surrounding active-site environment.

These structural differences provide a molecular explanation for the higher PA and GPB values predicted by the QM/MM calculations. The extensive hydrogen-bonding network, together with the favorable electrostatic interaction between the positively charged N-protonated centre and the negatively charged Glu93 residue, preferentially stabilizes the protonated S-O-NH crosslink. Collectively, the QM-cluster and QM/MM results indicate that protonation at the nitrogen centre is energetically preferred. In particular, the explicit protein environment, including the crystallographic water molecules, the surrounding hydrogen-bonding network, and the favorable electrostatic interaction between the positively charged N-protonated centre and the negatively charged Glu93 residue, further stabilizes the S-O-NH_2_^+^ crosslink. Although the QM/MM proton affinity and gas-phase basicity values should not be interpreted as a direct quantitative extension of the continuum QM-cluster calculations, they highlight the important role the protein environment in modulating the protonation energetics of the crosslink.

However, both the QM-cluster and QM/MM calculations provide only a static description of the active-site structure and the associated hydrogen-bonding interactions. They do not address whether the favourable hydrogen-bonding network surrounding the protonated crosslink is maintained under physiological thermal fluctuations or whether protonation influences the structural dynamics of the active site over time. To address these questions, MD simulations were performed for the uncrosslinked (Cys38-SH/Lys8-NH_3_^+^), neutral crosslinked (S-O-NH), and protonated crosslinked (S-O-NH_2_^+^) systems. The simulations were designed to evaluate the stability of the hydrogen-bonding interactions observed in the QM/MM optimized structures and the available crystal structures (PDB IDs: 7ODP, 7ODO, 6ZWJ, and 6ZWH),^1,2^ while also examining the effect of crosslink protonation on the conformational dynamics of the active site.

To determine whether the hydrogen-bonding interactions identified by the QM/MM calculations are maintained under dynamic conditions, hydrogen-bond networks between the S-O-NH/S-O-NH_2_^+^ crosslink and Glu93 were analysed over the final 100 ns of the MD simulations (Fig. 4). The protonated crosslink predominantly formed a direct hydrogen bond with Glu93, with a mean occupancy of approximately 69%, while one-water-bridged interactions accounted for a further ∼18%. In contrast, the neutral crosslink rarely formed a direct hydrogen bond and was predominantly found in conformations lacking any hydrogen-bonding interaction with Glu93 (∼66%). When interactions were present, they occurred mainly through two-water bridges (∼27%).

**Fig. 4:**
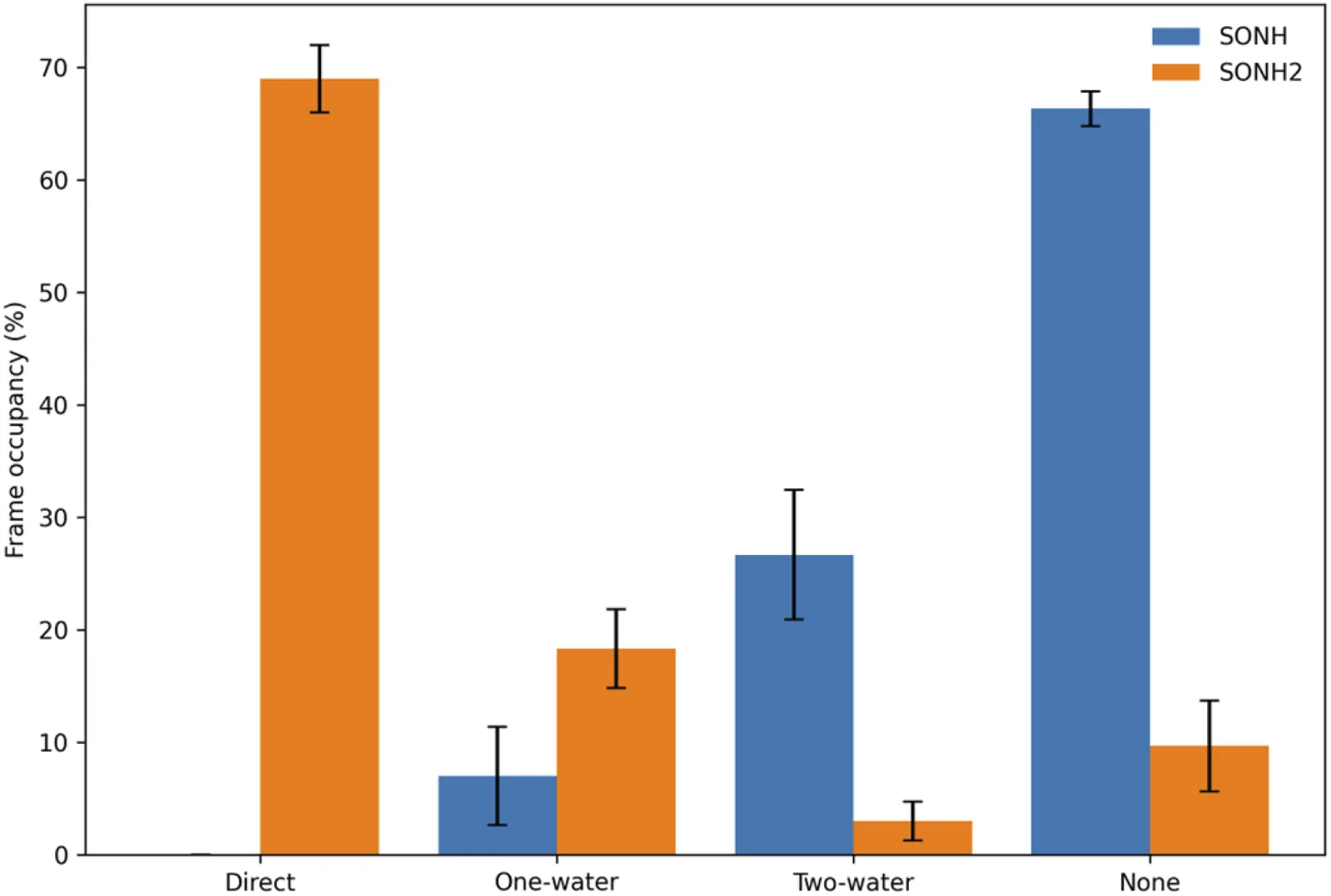
Occupancy of hydrogen-bonding interactions between the S-O-NH/S-O-NH_2_^+^ crosslink and Glu93 during the final 100 ns of the molecular dynamics simulations. Hydrogen-bond networks were classified as direct hydrogen bonds, one-water bridges, two-water bridges, or no interaction. Values represent the mean occupancy across three independent 200 ns simulations, and error bars denote the standard deviation.

These results demonstrate that protonation substantially strengthens the interaction between the crosslink and Glu93 by favoring persistent hydrogen-bonding interactions, either directly or through bridging water molecules. This dynamic behavior is consistent with the QM/MM optimized structures and complements the available crystal structures (PDB IDs: 7ODP, 6ZWJ, and 6ZWH),^1,2^ which capture a water-mediated interaction between the protonated crosslink nitrogen and Glu93 (average N⋯OE distance ≈ 4.7 Å). The MD simulations further indicate that this interaction is dynamic, interconverting between direct and water-mediated hydrogen-bonding arrangements while maintaining a persistent association between the protonated crosslink and Glu93. The corresponding time-evolution plots of the N⋯OE1, N⋯OE2, and minimum N⋯O distances between the protonated crosslink nitrogen and the Glu93 carboxylate oxygen atoms further support the persistence of these interactions throughout the simulations (Fig. S2). Collectively, these findings show that, when nitrogen protonation is prescribed, the S-O-NH_2_^+^ crosslink is preferentially stabilized by persistent interactions with Glu93 and the surrounding water network.

To further investigate the structural basis of the persistent hydrogen-bonding interactions with Glu93, the side-chain conformations of Glu93 were analyzed by clustering the *χ*_1_ and *χ*_2_ dihedral angles over the final 100 ns of the MD simulations (Fig. 5). The *χ*_1_/*χ*_2_ conformational maps reveal a pronounced protonation-dependent shift in the preferred side-chain orientation of Glu93. In the neutral SONH system, approximately 77% of the conformational ensemble occupies a gauche− *χ*_1_ rotamer centred around *χ*_1_ ≈ −58° and *χ*_2_ ≈ −50°. In contrast, protonation markedly redistributes the conformational ensemble, with approximately 73% of the S-O-NH_2_^+^ trajectory populating a trans *χ*_1_ rotamer centred around *χ*_1_ ≈ ±180° and *χ*_2_ ≈ +58°, while the population of the gauche− *χ*_1_ rotamer is substantially reduced. The populations located at *χ*_1_ ≈ +180° and *χ*_1_ ≈ −180° represent the same continuous rotamer because of the periodic nature of the dihedral angle. Importantly, this conformational transition is accompanied by a pronounced decrease in the average minimum crosslink N⋯O distance, from approximately 6.1 Å in the dominant S-O-NH conformations to 2.8 Å in the dominant S-O-NH_2_^+^ conformation. Collectively, these results indicate that nitrogen protonation shifts the conformational equilibrium of the Glu93 side chain towards a rotameric state that positions the carboxylate closer to the crosslink, thereby providing a structural basis for the enhanced hydrogen-bond occupancy and stabilization observed for the protonated S-O-NH_2_^+^ crosslink.

**Fig. 5:**
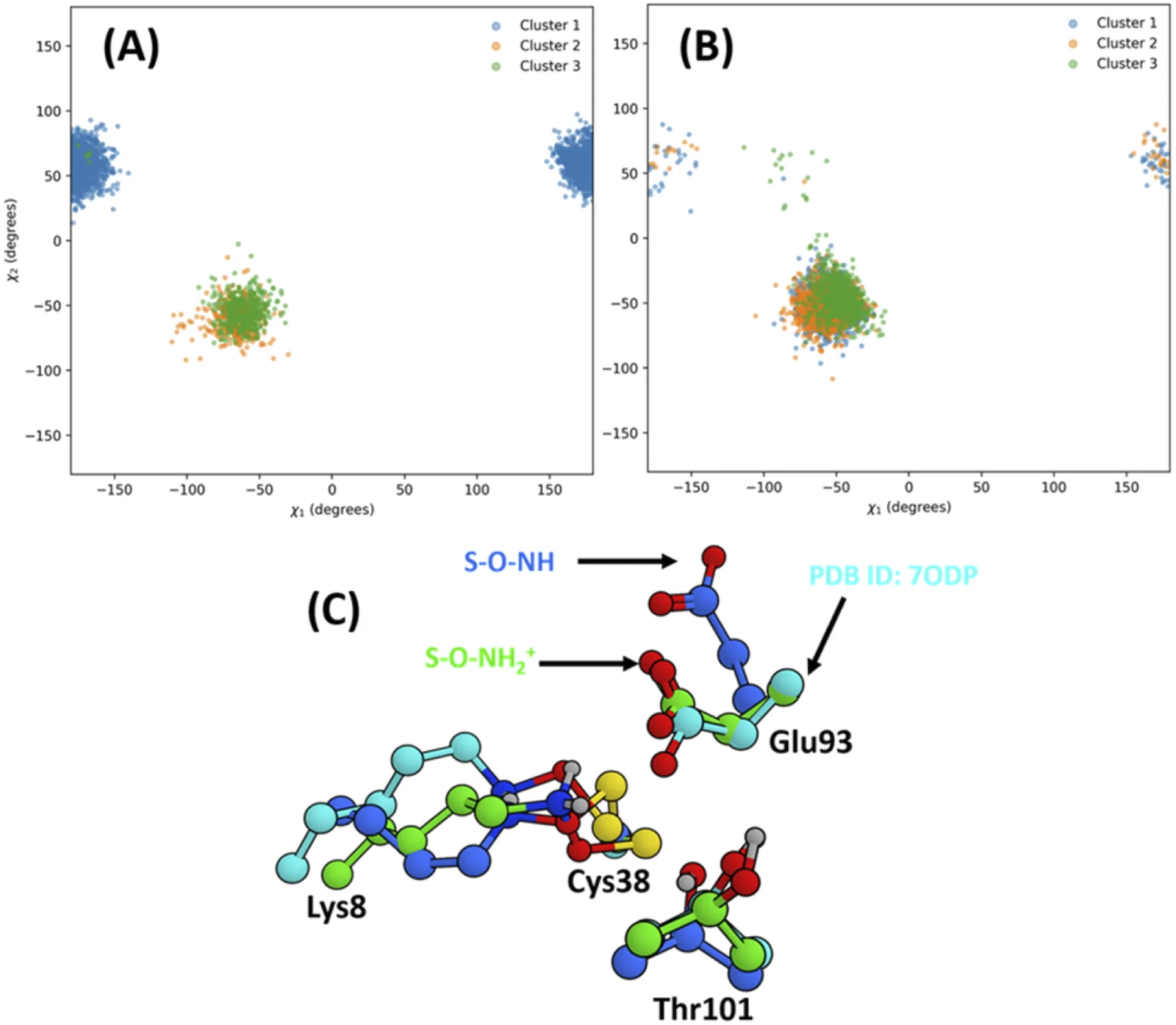
Conformational clustering of the Glu93 side chain based on the *χ*_1_ and *χ*_2_ dihedral angles during the final 100 ns of the molecular dynamics simulations for the (A) protonated S-O-NH_2_^+^ and (B) neutral S-O-NH crosslinked systems. (C) Superposition of the most populated cluster from each simulation with the crystal structure (PDB ID: 7ODP). The protonated S-O-NH_2_^+^ crosslink adopts a Glu93 conformation that closely resembles the crystallographic structure and favors hydrogen-bond formation with the crosslink.

Superposition of the most populated clusters further illustrates this difference (Fig. 5C). In the protonated system, the dominant Glu93 conformation closely matches that observed in the crystal structure (PDB ID: 7ODP), positioning the carboxylate group toward the protonated crosslink to facilitate hydrogen-bond formation. Conversely, the neutral system adopts a less favorable orientation, increasing the separation between the crosslink and Glu93. These observations are consistent with the QM/MM optimized structures and provide additional structural evidence that protonation preferentially stabilizes the S-O-NH_2_^+^ crosslink by promoting a persistent hydrogen-bonding interaction with Glu93. Collectively, the hydrogen-bond occupancy, distance, and conformational clustering analyses demonstrate that nitrogen protonation strengthens the interaction between the crosslink and Glu93. In fact, protonation also favors a preferred Glu93 side-chain orientation that closely resembles the experimentally observed active-site architecture.

Since our results support the N-protonated S-O-NH_2_^+^ species as the most plausible form of the crosslink, we next investigated whether its protonation state influences the global conformational dynamics of the protein. The backbone RMSD profiles stabilized rapidly and remained relatively consistent throughout the final 100 ns of the simulations. The reported RMSD values represent averages across the three independent replicate simulations (Fig. 6), while the replicate-specific analyses are provided in the SI (Fig. S5).

**Fig. 6:**
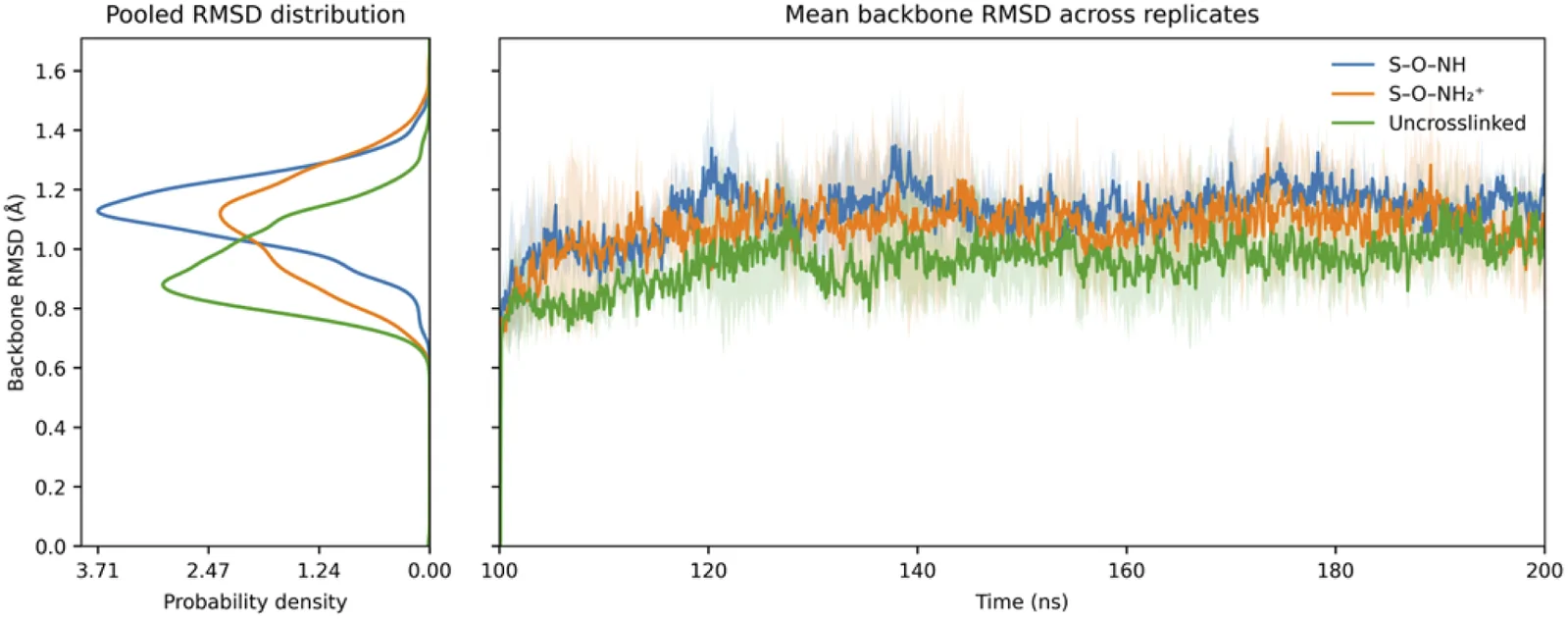
Mean backbone RMSD for structures containing neutral (S-O-NH; blue) and protonated (S-O-NH_2_^+^; orange) crosslinks and the uncrosslinked (Cys38 as a thiol; green) systems, relative to their initial starting positions. The right panel shows the time evolution of the backbone RMSD during the final 100 ns of the simulation, calculated relative to the respective starting structure, while the left panel shows the corresponding RMSD probability-density distribution.

Despite their overall structural stability, the three systems exhibited distinct conformational behavior. The uncrosslinked protein had the lowest average backbone RMSD (0.954 ± 0.135 Å), indicating the smallest deviation from its starting structure. In contrast, both crosslinked systems sampled conformations with larger backbone deviations. This behavior is consistent with subtle structural rearrangements following formation of the crosslink. Among the crosslinked systems, the neutral S-O-NH state exhibited the highest average RMSD (1.124 ± 0.125 Å), whereas the protonated S-O-NH_2_^+^ state displayed a slightly lower value (1.073 ± 0.167 Å).

The corresponding RMSD distributions showed the same overall trend. The uncrosslinked protein exhibited a greater population at lower RMSD values, whereas the distribution for the neutral S-O-NH system was shifted towards higher values. The protonated S-O-NH_2_^+^ system displayed an intermediate distribution. Collectively, these results indicate that both crosslink formation and nitrogen protonation influence the global conformational dynamics of the protein. Although the overall differences are modest, the distinct behavior of the neutral and protonated systems suggests that the protonation state contributes to the conformational landscape. Therefore, nitrogen protonation should be explicitly considered in future structural and mechanistic studies of S-O-NH crosslinks.

## 4. Conclusion

In the present study, the protonation state of the putative Cys38-S-O-NH-Lys8 crosslink in transaldolase from *Neisseria gonorrhoeae* (NgTAL) was investigated using complementary QM-cluster, QM/MM, and MD simulations. Collectively, these approaches provide consistent evidence that the N-protonated S-O-NH_2_^+^ species is the most plausible form of the crosslink, rather than the proposed neutral S-O-NH species.

The QM-cluster calculations showed that the nitrogen centre has the highest PA and GPB across the gas-phase (*ε* = 1.0), protein (*ε* = 4.0), and aqueous (*ε* = 78.3) dielectric environments considered. Moreover, explicit inclusion of the three crystallographic water molecules provided a proton-transfer pathway to the crosslink nitrogen, while no stable S- or O-protonated species was obtained. The QM/MM calculations similarly favored nitrogen protonation, yielding PA and GPB values of 1700.2 and 1685.5 kJ mol^−1^, respectively. Although these values should not be directly compared quantitatively with those obtained from the continuum QM-cluster models, they demonstrate the substantial influence of the explicitly represented protein environment on the protonation energetics. In particular, the crystallographic water molecules and Glu93 form an extended hydrogen-bonding network that preferentially stabilizes the positively charged N-protonated crosslink.

The MD simulations further demonstrated that this interaction persists under dynamic conditions. The protonated S-O-NH_2_^+^ crosslink formed a direct hydrogen bond with Glu93 with a mean occupancy of approximately 69%, while one-water-bridged interactions accounted for a further ∼18%. In contrast, the neutral S-O-NH crosslink predominantly occupied conformations lacking a hydrogen-bonding interaction with Glu93 (∼66%) and interacted mainly through less persistent two-water bridges when an association was present. Nitrogen protonation also shifted the conformational equilibrium of Glu93 towards a side-chain orientation that positions its carboxylate closer to the crosslink and closely resembles the experimentally observed active-site architecture. Although all three MD systems remained structurally stable and the differences in backbone RMSD were modest, the neutral crosslinked system exhibited the largest average deviation from its starting structure, while the protonated system displayed intermediate behavior between the neutral and uncrosslinked systems. Thus, nitrogen protonation primarily stabilizes the local crosslink environment through persistent interactions with Glu93 and surrounding water molecules, while also contributing to the global conformational landscape of the protein. These findings establish the N-protonated S-O-NH_2_^+^ crosslink as the most plausible form in NgTAL and provide a foundation for future investigations into the formation, stability, and biological roles of such crosslinks in proteins. In particular, comparative studies of NgTAL and the homologous transaldolase from *Neisseria meningitidis* (NmTAL) could investigate whether differences in the local electrostatic environment alter the protonation state of the crosslink nitrogen, potentially contributing to the observed differences in their responses to crosslink formation.^31^

## Author contributions

The manuscript was written through contributions of all authors. All authors have given approval to the final version of the manuscript.

## Conflicts of interest

There are no conflicts to declare.

## Supplementary Material

RA-016-D6RA07624F-s001

## Data Availability

The analysis scripts used for the molecular dynamics trajectory analyses, including the hydrogen-bond network, crosslink-Glu93 distance, Glu93 conformational clustering, and backbone RMSD analyses, are publicly available through Zenodo at https://doi.org/10.5281/zenodo.21612828. The deposited files also include the AMBER topology and coordinate files used for the neutral S-O-NH, N-protonated S-O-NH_2_^+^, and uncrosslinked NgTAL simulations, together with the optimized QM/MM structures reported in this study. Supplementary information (SI): QM-cluster models and method-assessment data; additional crosslink-Glu93 distance, hydrogen-bonding, conformational-clustering, and backbone RMSD analyses; RESP parameterization procedures and AMBER force-field parameters; structural validation data; and a representative MD input file. See DOI: https://doi.org/10.1039/d6ra07624f.
