# Supplementary material for: Protonation state of the S-O-NH crosslink between lysine and cysteine
Source: RSC Adv. 2026 Sep 22;16(45):56915–23. doi: 10.1039/d6ra07624f (PMC13595585; doi:10.1039/d6ra07624f)
Supplement: Supplementary file 1 [file RA-016-D6RA07624F-s001.pdf]

Supplementary Information (SI) for RSC Advances.
This journal is © The Royal Society of Chemistry 2026

Supporting Information

Protonation State of the -SONH- Cross-link Between Lysine and Cysteine

Danajana V. Wijerathne, [a,b] Michaela A. Morris,[a] James W. Gauld[a,b *]

[a] Department of Chemistry and Biochemistry, University of Windsor, Windsor Ontario, N9B 3P4, Canada

[b] Department of Chemistry, Memorial University of Newfoundland, St. John's NL, A1C 5S7, Canada

Total pages: 16
(Computational Methods, Figures S1-S5; Tables S1-S5)

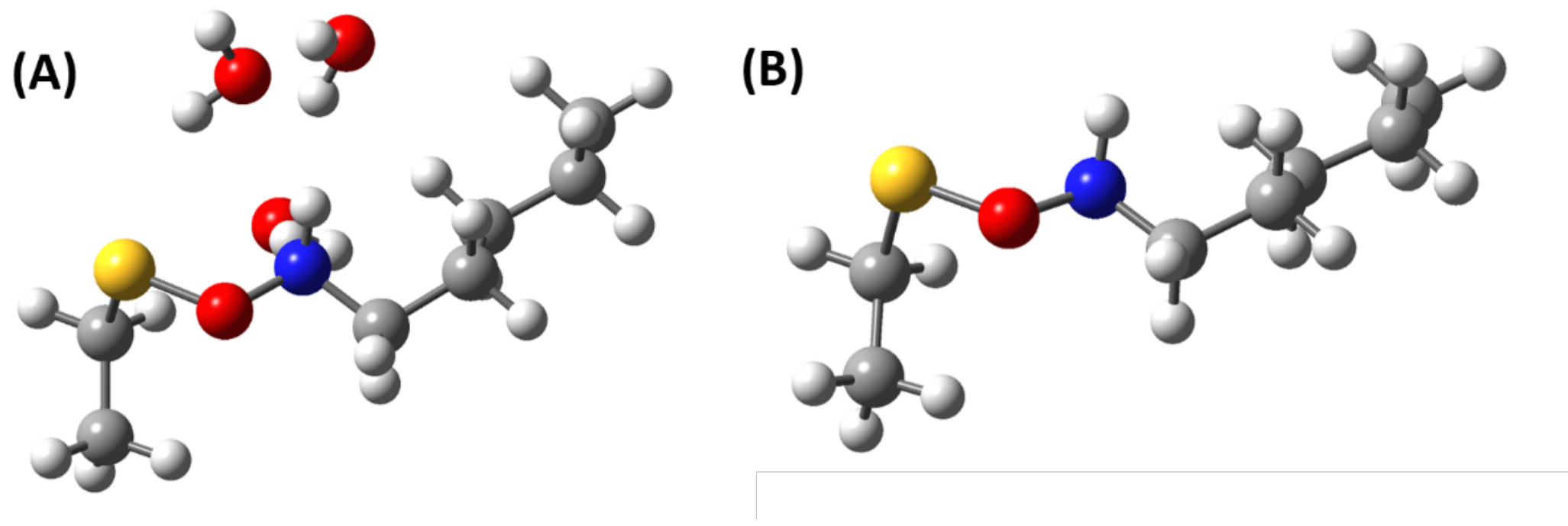

Figure S1. QM-cluster models used for the PA and GPB calculations. (A) QM-cluster model including the three crystallographic water molecules (B) QM-cluster model without the crystallographic water molecules.

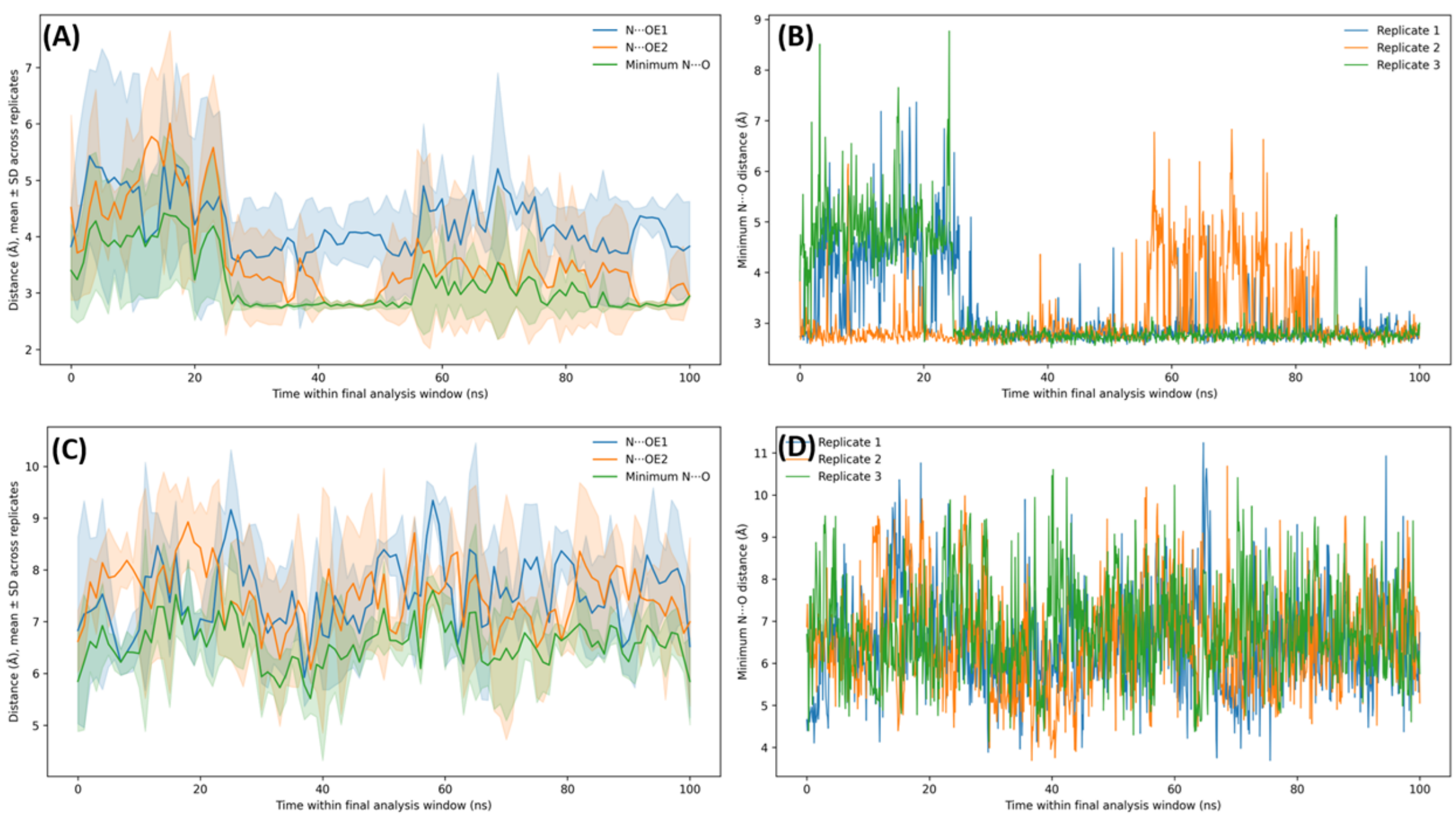

Figure S2. Effect of crosslink nitrogen protonation on the interaction between the crosslink and Glu93. The distances between the crosslink nitrogen atom and the two carboxylate oxygen atoms of Glu93 (N···OE1 and N···OE2) were monitored during the final 100 ns of each MD simulation. The minimum N···O distance was defined as the shorter of the N···OE1 and N···OE2 distances at each time point, thereby accounting for dynamic switching of the interaction between the two carboxylate oxygen atoms. (A, C) Mean N···OE1, N···OE2, and minimum N···O distances for the protonated S-O-NH_2^+ (A) and neutral S-O-NH (C) systems. Distances were averaged in 1 ns bins; solid lines represent the mean and shaded regions indicate ± standard deviation across three independent replicate simulations. (B, D) Minimum N···O distance profiles for the three individual

replicates of the S-O-NH_2^+ (B) and S-O-NH (D) systems, respectively. All distances are reported in Å.

System	Replicate	Direct	One Water	Two Water	None
SONH	1	0	5	30	65
SONH	2	0	12	20	68
SONH	3	0	4	30	66
SONH2	1	66	18	4	12
SONH2	2	72	22	1	5
SONH2	3	69	15	4	12

Figure S3. % Occupancies of direct and water-mediated hydrogen-bonding networks between the crosslink and Glu93. Each analyzed trajectory frame was assigned to one of four mutually exclusive categories: a direct hydrogen bond, a one-water bridge, a two-water bridge, or no hydrogen-bonding interaction ("None"). Occupancies are reported as the percentage of analyzed frames for each of the three independent replicate simulations of the neutral S-O-NH and protonated S-O-NH_2^+ systems. The overall hydrogen-bond network occupancy corresponds to the sum of the direct, one-water-bridge, and two-water-bridge occupancies (equivalent to 100% minus the "None" occupancy).

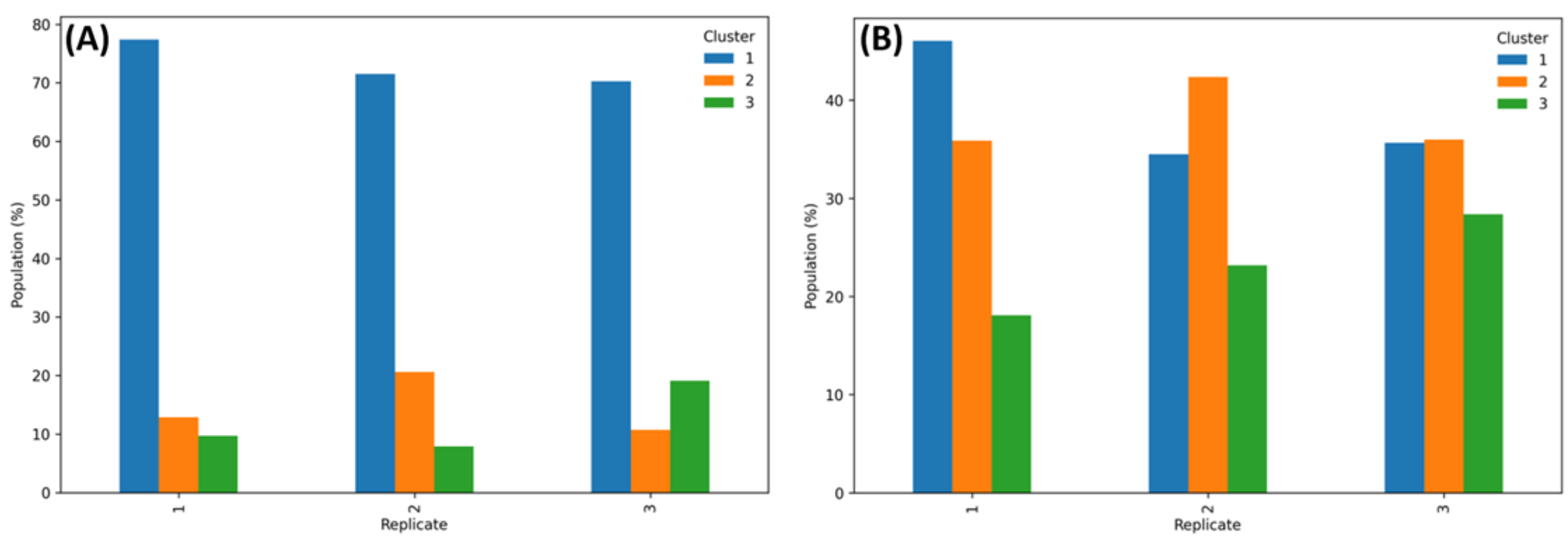

Figure S4. Population distributions of the Glu93 conformational clusters across the three independent MD replicates. Conformational clustering was performed using the final 100 ns of each simulation based on the Glu93 χ_1 (N–Cα–Cβ–Cγ) and χ_2 (Cα–Cβ–Cγ–Cδ) dihedral angles and the N···OE1 and N···OE2 distances between the crosslink nitrogen and the Glu93 carboxylate oxygen atoms. The sine and cosine components of χ_1 and χ_2 were used to account for dihedral periodicity. Bars show the percentage of trajectory frames assigned to each of the three clusters for the (A) protonated S-O-NH_2^+ and (B) neutral S-O-NH systems.

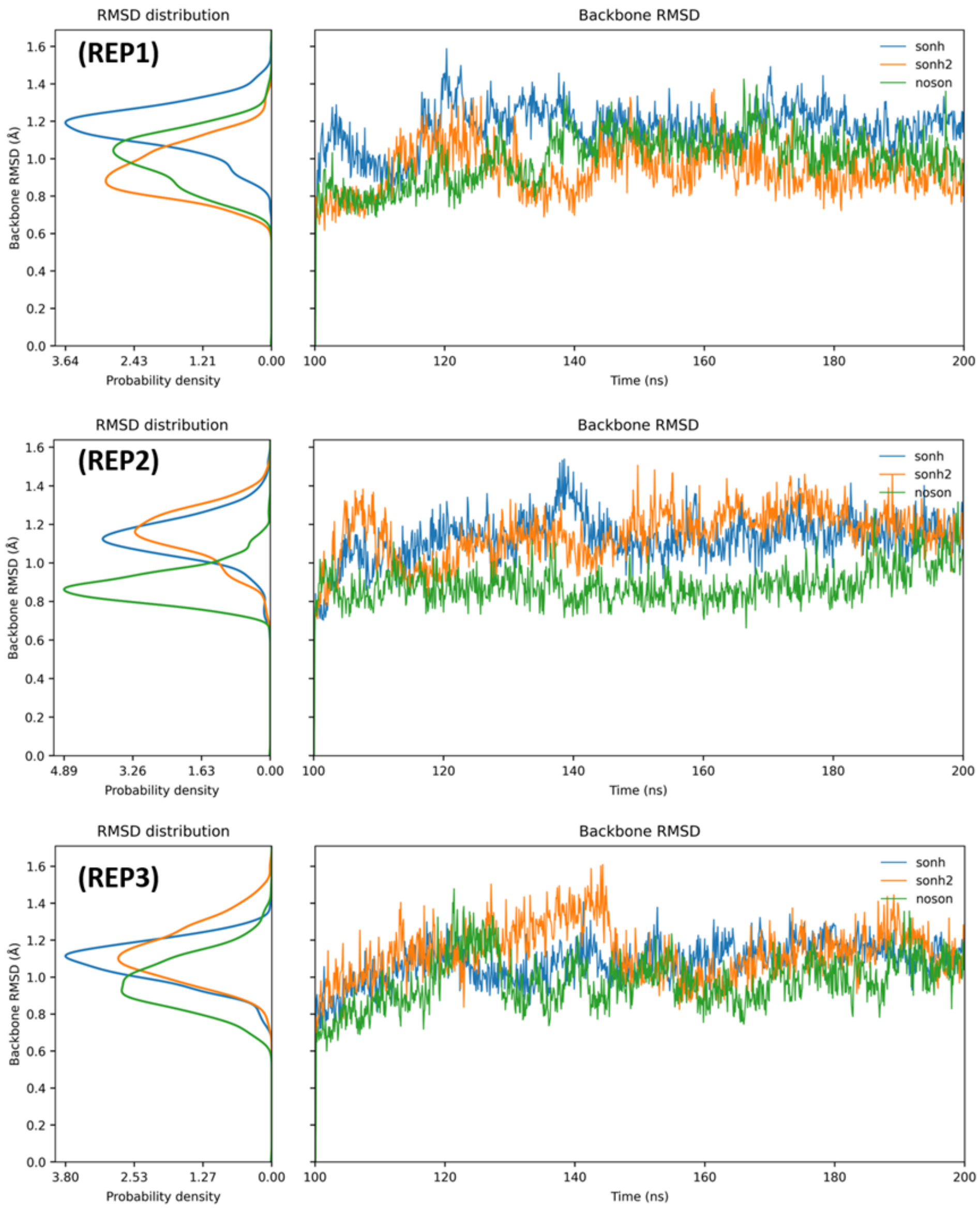

Figure S5. Backbone RMSD profiles and corresponding distributions for the three independent MD replicates. For each replicate (REP1-REP3), the right panel shows the time evolution of the backbone RMSD during the final 100 ns of the simulation, calculated relative to the respective starting structure, while the left panel shows the corresponding RMSD probability-density distribution. Results are presented for structures containing the neutral S-O-NH crosslink (blue), the protonated S-O-NH_2^+ crosslink (orange), and the uncrosslinked Cys38 thiol (green). RMSD values are reported in Å.

Table S1. Selected optimized bond lengths (Å), bond angles (degrees), proton affinities (PA), and gas-phase basicities (GPB) (kJ mol^{-1}) obtained from the method assessment using the CH_3CH_2-S-O-NH-CH_2CH_3 model.

	Basis set					
**DFT Functional**		6-311G	6-311G(d,p)	6-311G(2d,2p)	6-311++G(d,p)	6-311++G(2d,2p)
**M06-2X**	S-O	1.804	1.677	1.665	1.677	1.666
	N-O	1.459	1.434	1.438	1.432	1.436
	<SON	110.1	111.4	111.0	111.7	111.3
	PA	903.7	896.0	901.2	892.4	897.0
	GPB	873.7	865.1	870.3	861.3	865.8
**B3LYP-D3**	S-O	1.842	1.695	1.681	1.696	1.682
	N-O	1.480	1.460	1.465	1.458	1.463
	<SON	110.2	111.5	111.2	112.0	111.6
	PA	918.0	913.5	917.9	907.3	911.5
	GPB	888.0	884.2	889.2	877.8	882.4
**ωB97X-D**	S-O	1.817	1.678	1.665	1.679	1.666
	N-O	1.455	1.436	1.439	1.434	1.438
	<SON	110.5	111.7	111.3	112.1	111.7
	PA	920.4	913.8	920.9	910.8	915.7
	GPB	890.5	883.4	892.1	881.1	886.5

CCSD/6-311G(d,p)	S-O	1.684
	N-O	1.443
	<SON	111.0
	PA	919.6
	GPB	889.6

Table S2. Computational workflow used to generate RESP-derived atomic charges and AMBER force-field parameters for the neutral (-S-O-NH-) and protonated (-S–O–NH_2^+-) Lys8-Cys38 crosslinked residues.

The neutral (-S-O-NH-) and protonated (-S-O-NH_2^+-) crosslinked Lys8-Cys38 residues were parameterized using the RESP protocol implemented in AmberTools to ensure compatibility with the AMBER force field. The complete workflow is summarized below. In addition, the complete AMBER topology and coordinate files used for the S-O-NH, S-O-NH_2^+, and uncrosslinked simulations are available at https://doi.org/10.5281/zenodo.21612828. The repository also contains the analysis scripts used to generate the hydrogen-bond network, crosslink-Glu93 distance, Glu93 conformational clustering, and backbone RMSD results presented in the manuscript and Supporting Information.

Step 1. Construction of the crosslink models

The crosslinked Lys8 and Cys38 residues were extracted from the crystal structure of the S-O-NH crosslinked transaldolase (PDB ID: 7ODP). The peptide backbone was truncated and capped to generate a reduced model suitable for quantum chemical calculations. Heavy atoms were restrained during the initial optimization to preserve the crystallographic geometry of the crosslink. Separate protonated (A) and neutral (B) models were then constructed as shown below.

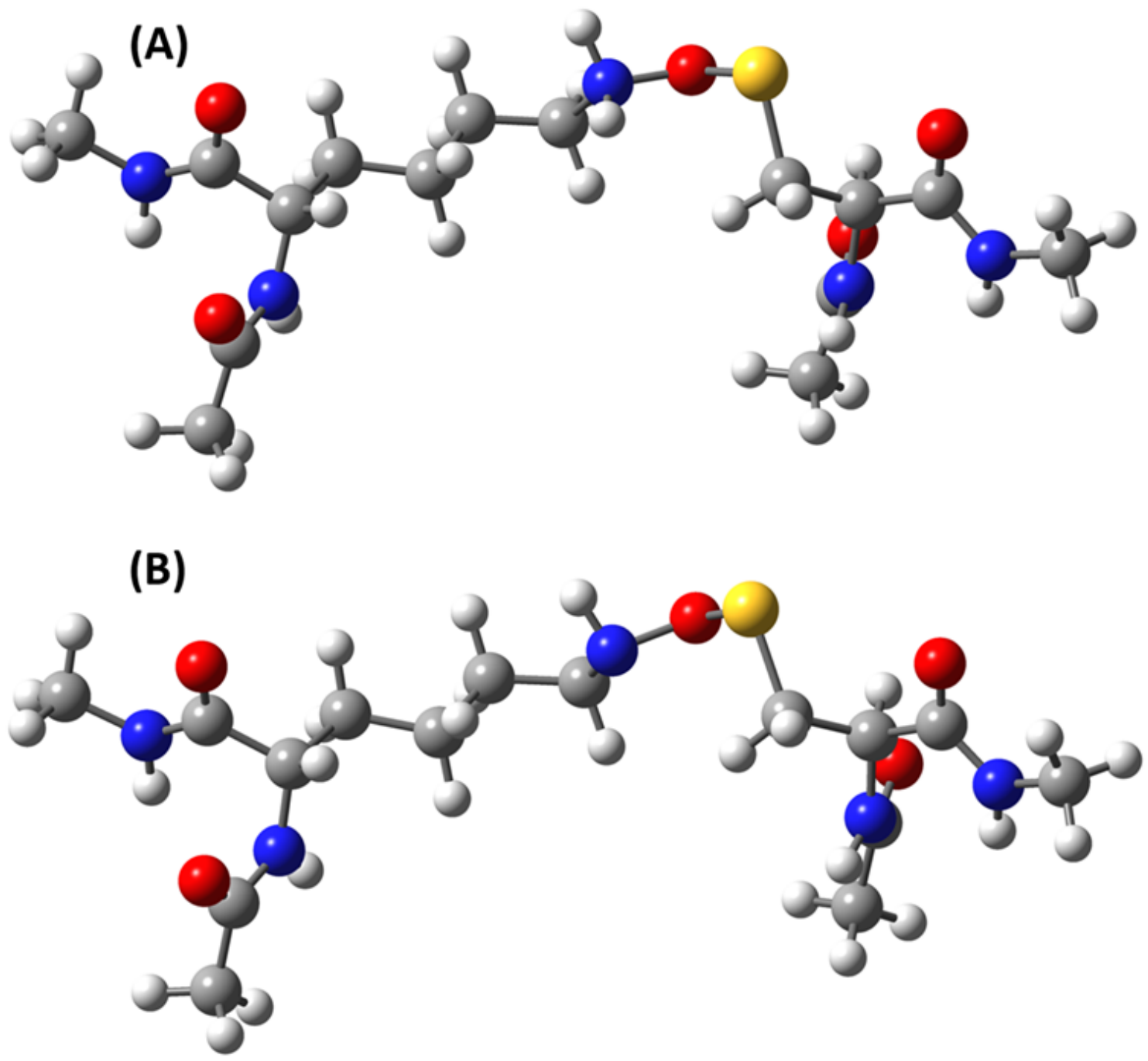

Step 2. Quantum mechanical geometry optimization

The neutral and protonated crosslink models were optimized using Gaussian 16 at the ωB97X-D/6-311G(2d,2p) level of theory. The optimized structures were subsequently used for electrostatic potential (ESP) calculations.

Step 3. Electrostatic potential calculations

Following geometry optimization, single-point ESP calculations were performed on the optimized neutral (-S-O-NH-) and protonated (-S-O-NH_2^+-) crosslink models using HF/6-31G(d) in Gaussian 16.

Step 4. Generation of RESP charges

The molecular electrostatic potential calculated from the optimized Gaussian wavefunction was converted into an ESP file using:

espgen -i crosslink.log -o esp-file.esp

An Amber AC file was generated from the Gaussian output:

antechamber -i crosslink.log -fi gout -o ac-file.ac -fo ac -c resp -nc 1

where -nc 1 specifies the total charge of the protonated crosslink (the neutral model was assigned the corresponding neutral charge).

The RESP input files for the two-stage fitting procedure were generated using:

```
respgen -i ac-file.ac -o stage1.respin1 -f resp1
respgen -i ac-file.ac -o stage2.respin2 -f resp2
```

The two-stage RESP fitting was then performed using:

```
resp -O -i stage1.respin1 -o stage1.respout1 \
-e esp-file.esp -t qout-stage1

resp -O -i stage2.respin2 -o stage2.respout2 \
-e esp-file.esp -q qout-stage1 -t qout-stage2
```

The resulting RESP charges (qout-stage2) were used for all subsequent force-field parameterization.

Step 5. Construction of RESP MOL2 files

A preliminary MOL2 file was first generated:

```
antechamber \
-i crosslink.log \
-fi gout \
-o crosslink_tmp.mol2 \
-fo mol2 \
-at gaff2 \
-c bcc
```

The temporary AM1-BCC charges were then replaced with the RESP-derived charges:

```
antechamber \
-i crosslink_tmp.mol2 \
-fi mol2 \
-o crosslink_resp.mol2 \
-fo mol2 \
-c rc \
-cf qout-stage2 \
-at gaff2
```

Finally, the RESP-parameterized residue was converted to use standard AMBER atom types:

```
antechamber \
-i crosslink_resp.mol2 \
-fi mol2 \
-o amber-atom-type.mol2 \
-fo mol2 \
```

-at amber

Step 6. Force-field parameter generation

The force-field modification file (crosslink.frcmod) was generated using Parmchk2 in AmberTools. The RESP-derived MOL2 file containing AMBER atom types was used as the input, and the General AMBER Force Field (GAFF2) parameter database was employed to assign the bonded parameters for the modified S-O-NH crosslinked residues:

```
parmchk2 \
-i amber-atom-type.mol2 \
-f mol2 \
-o crosslink.frcmod \
-p $AMBERHOME/dat/leap/parm/gaff2.dat
```

The generated crosslink.frcmod file contained all the required bonded parameters for the modified residues, with no missing parameters identified by Parmchk2. This force-field modification file was subsequently used together with the RESP-derived residue library (.lib) during system preparation for the molecular dynamics simulations.

Table S3 Generated AMBER residue force-field parameter files for the crosslinked residues.

(a) Neutral S-O-NH crosslink

Force-field modification file (crosslink_neutral.frcmod)

```
Remark line goes here
MASS
CT 12.010        0.878              same as c3
C  12.010        0.616              same as c
O  16.000        0.434              same as o
HC 1.008         0.135              same as hc
N  14.010        0.530              same as n
NT 14.010        0.530              same as n3
H  1.008         0.161              same as hn
H1 1.008         0.135              same as h1
S  32.060        2.900              same as ss
OS 16.000        0.465              same as os

BOND
C -CT  234.85   1.527       same as  c-c3, penalty score=  0.0
CT-HC  345.25   1.096       same as c3-hc, penalty score=  0.0
C -O   590.60   1.219       same as  c- o, penalty score=  0.0
C -N   317.46   1.397       same as  c- n, penalty score=  0.0
CT-N   254.46   1.464       same as c3- n, penalty score=  0.0
H -N   527.30   1.013       same as hn- n, penalty score=  0.0
```

CT-CT 228.89   1.535   same as c3-c3, penalty score= 0.0
CT-H1 344.22   1.097   same as c3-h1, penalty score= 0.0
CT-NT 255.85   1.463   same as c3-n3, penalty score= 0.0
H -NT 511.30   1.019   same as hn-n3, penalty score= 0.0
NT-OS 166.83   1.450   same as n3-os, penalty score= 0.0
CT-S   184.74   1.841   same as c3-ss, penalty score= 0.0
OS-S   299.81   1.664   same as os-ss, penalty score= 0.0

ANGLE
CT-C -O   76.450   122.900   same as c3-c -o , penalty score= 0.0
CT-C -N   65.190   117.160   same as c3-c -n , penalty score= 0.0
C -CT-HC   43.630   108.950   same as c -c3-hc, penalty score= 0.0
C -N -CT   77.240   118.830   same as c -n -c3, penalty score= 0.0
C -N -H   48.700   117.550   same as c -n -hn, penalty score= 0.0
N -C -O   86.680   122.570   same as n -c -o , penalty score= 0.0
HC-CT-HC   35.800   107.730   same as hc-c3-hc, penalty score= 0.0
C -CT-N   65.390   111.940   same as c -c3-n , penalty score= 0.0
CT-CT-N   64.950   112.780   same as c3-c3-n , penalty score= 0.0
H1-CT-N   48.090   108.760   same as h1-c3-n , penalty score= 0.0
CT-N -H   46.100   117.680   same as c3-n -hn, penalty score= 0.0
CT-CT-CT   59.870   112.630   same as c3-c3-c3, penalty score= 0.0
CT-CT-HC   43.270   109.680   same as c3-c3-hc, penalty score= 0.0
C -CT-CT   60.360   111.430   same as c -c3-c3, penalty score= 0.0
C -CT-H1   43.740   108.370   same as c -c3-h1, penalty score= 0.0
CT-CT-H1   43.280   109.590   same as c3-c3-h1, penalty score= 0.0
CT-CT-NT   64.900   113.080   same as c3-c3-n3, penalty score= 0.0
CT-NT-H   47.800   109.290   same as c3-n3-hn, penalty score= 0.0
CT-NT-OS   98.460   105.590   same as c3-n3-os, penalty score= 0.0
H1-CT-NT   47.840   110.090   same as h1-c3-n3, penalty score= 0.0
NT-OS-S   101.100   112.458   same as na-os-ss, penalty score= 0.0
H1-CT-H1   35.640   108.550   same as h1-c3-h1, penalty score= 0.0
H -NT-OS   62.700   102.750   same as hn-n3-os, penalty score= 0.0
CT-CT-S   62.920   110.180   same as c3-c3-ss, penalty score= 0.0
CT-S -OS 116.850   100.300   same as c3-ss-os, penalty score= 0.0
H1-CT-S   42.060   108.730   same as h1-c3-ss, penalty score= 0.0

DIHE
CT-C -N -CT   1   1.410   180.000   -2.000   same as c3-c -n -c3
CT-C -N -CT   1   2.930   0.000   -1.000   same as c3-c -n -c3
CT-C -N -CT   1   0.500   0.000   3.000   same as c3-c -n -c3, penalty score= 0.0
CT-C -N -H   4   10.000   180.000   2.000   same as X -c -n -X , penalty score= 0.0
C -CT-N -C   1   0.390   180.000   -2.000   same as c -c3-n -c
C -CT-N -C   1   0.640   0.000   1.000   same as c -c3-n -c , penalty score= 0.0
CT-CT-N -C   1   0.650   180.000   -4.000   same as c3-c3-n -c
CT-CT-N -C   1   0.030   180.000   -3.000   same as c3-c3-n -c
CT-CT-N -C   1   2.260   0.000   1.000   same as c3-c3-n -c , penalty score= 0.0

H1-CT-N -C	1	0.000	180.000	2.000	same as h1-c3-n -c , penalty score= 0.0
O -C -N -CT	4	10.000	180.000	2.000	same as X -c -n -X , penalty score= 0.0
O -C -N -H	1	2.500	180.000	-2.000	same as o -c -n -hn
O -C -N -H	1	2.000	0.000	1.000	same as o -c -n -hn, penalty score= 0.0
O -C -CT-HC	1	4.030	0.000	-1.000	same as o -c -c3-hc
O -C -CT-HC	1	3.660	180.000	2.000	same as o -c -c3-hc, penalty score= 0.0
N -C -CT-HC	6	0.000	180.000	2.000	same as X -c -c3-X , penalty score= 0.0
O -C -CT-N	6	0.000	180.000	2.000	same as X -c -c3-X , penalty score= 0.0
N -C -CT-N	1	0.100	180.000	-1.000	same as n -c -c3-n
N -C -CT-N	1	2.120	180.000	2.000	same as n -c -c3-n , penalty score= 0.0
CT-CT-CT-N	1	0.100	0.000	3.000	same as c3-c3-c3-n , penalty score= 0.0
HC-CT-CT-N	9	1.400	0.000	3.000	same as X -c3-c3-X , penalty score= 0.0
CT-CT-CT-CT	1	0.520	0.000	3.000	same as c3-c3-c3-c3, penalty score= 0.0
CT-CT-CT-HC	1	0.130	0.000	3.000	same as c3-c3-c3-hc, penalty score= 0.0
C -CT-CT-CT	1	0.000	0.000	-3.000	same as c -c3-c3-c3
C -CT-CT-CT	1	0.040	0.000	-2.000	same as c -c3-c3-c3
C -CT-CT-CT	1	0.460	180.000	1.000	same as c -c3-c3-c3, penalty score= 0.0
C -CT-CT-HC	9	1.400	0.000	3.000	same as X -c3-c3-X , penalty score= 0.0
O -C -CT-CT	1	0.200	180.000	-2.000	same as o -c -c3-c3
O -C -CT-CT	1	0.440	180.000	-3.000	same as o -c -c3-c3
O -C -CT-CT	1	0.680	0.000	1.000	same as o -c -c3-c3, penalty score= 0.0
N -C -CT-CT	1	0.000	180.000	-4.000	same as n -c -c3-c3
N -C -CT-CT	1	0.710	180.000	2.000	same as n -c -c3-c3, penalty score= 0.0
CT-CT-CT-NT	1	0.210	0.000	3.000	same as c3-c3-c3-n3, penalty score= 0.0
CT-CT-CT-H1	9	1.400	0.000	3.000	same as X -c3-c3-X , penalty score= 0.0
CT-CT-NT-H	1	0.217	0.000	3.000	same as c3-c3-n3-hn, penalty score= 0.0
CT-CT-NT-OS	6	1.800	0.000	3.000	same as X -c3-n3-X , penalty score= 0.0
CT-NT-OS-S	1	1.800	0.000	2.000	same as X -n3-os-X , penalty score= 0.0
C -CT-N -H	6	0.000	0.000	2.000	same as X -c3-n -X , penalty score= 0.0
CT-CT-N -H	6	0.000	0.000	2.000	same as X -c3-n -X , penalty score= 0.0
H1-CT-N -H	6	0.000	0.000	2.000	same as X -c3-n -X , penalty score= 0.0
O -C -CT-H1	1	0.800	0.000	-1.000	same as o -c -c3-h1
O -C -CT-H1	1	0.080	180.000	3.000	same as o -c -c3-h1, penalty score= 0.0
N -C -CT-H1	6	0.000	180.000	2.000	same as X -c -c3-X , penalty score= 0.0
H1-CT-CT-HC	9	1.400	0.000	3.000	same as X -c3-c3-X , penalty score= 0.0
HC-CT-CT-HC	1	0.120	0.000	3.000	same as hc-c3-c3-hc, penalty score= 0.0
HC-CT-CT-NT	1	0.100	0.000	3.000	same as hc-c3-c3-n3, penalty score= 0.0
H1-CT-NT-H	6	1.800	0.000	3.000	same as X -c3-n3-X , penalty score= 0.0
H1-CT-NT-OS	6	1.800	0.000	3.000	same as X -c3-n3-X , penalty score= 0.0
H -NT-OS-S	1	1.800	0.000	2.000	same as X -n3-os-X , penalty score= 0.0
N -CT-CT-S	9	1.400	0.000	3.000	same as X -c3-c3-X , penalty score= 0.0
H1-CT-CT-N	9	1.400	0.000	3.000	same as X -c3-c3-X , penalty score= 0.0
CT-CT-S -OS	3	1.000	0.000	3.000	same as X -c3-ss-X , penalty score= 0.0
NT-OS-S -CT	1	2.200	0.000	2.000	same as X -os-ss-X , penalty score= 0.0
C -CT-CT-S	9	1.400	0.000	3.000	same as X -c3-c3-X , penalty score= 0.0
C -CT-CT-H1	9	1.400	0.000	3.000	same as X -c3-c3-X , penalty score= 0.0

```
H1-CT-CT-S    9    1.400        0.000          3.000      same as X -c3-c3-X , penalty score=  0.0
H1-CT-CT-H1   9    1.400        0.000          3.000      same as X -c3-c3-X , penalty score=  0.0
H1-CT-S -OS   3    1.000        0.000          3.000      same as X -c3-ss-X , penalty score=  0.0

IMPROPER
CT-N -C -O        1.1         180.0        2.0         Using the default value
C -CT-N -H        1.1         180.0        2.0         Same as X -X -n -hn, penalty score=  6.0 (use
general term))

NONBON
  CT        1.9069  0.1078          same as c3
  C         1.8606  0.0988          same as c
  O         1.7107  0.1463          same as o
  HC        1.4593  0.0208          same as hc
  N         1.7852  0.1636          same as n
  NT        1.8886  0.0858          same as n3
  H         0.6210  0.0100          same as hn
  H1        1.3593  0.0208          same as h1
  S         1.9825  0.2824          same as ss
 OS         1.7713  0.0726          same as os
```

Force-field modification file (crosslink_protonated.frcmod)

```
Remark line goes here
MASS
CT 12.010       0.878           same as c3
C  12.010       0.616           same as c
O  16.000       0.434           same as o
HC 1.008        0.135           same as hc
N  14.010       0.530           same as n
N3 14.010       0.530           same as n4
H  1.008        0.161           same as hn
H1 1.008        0.135           same as h1
HP 1.008        0.135           same as hp
S  32.060       2.900           same as ss
OS 16.000       0.465           same as os

BOND
C -CT  313.00   1.524      same as  c-c3, penalty score=  0.0
CT-HC  330.60   1.097      same as c3-hc, penalty score=  0.0
C -O   637.70   1.218      same as  c- o, penalty score=  0.0
C -N   427.60   1.379      same as  c- n, penalty score=  0.0
CT-N   328.70   1.462      same as c3- n, penalty score=  0.0
H -N   403.20   1.013      same as hn- n, penalty score=  0.0
CT-CT  300.90   1.538      same as c3-c3, penalty score=  0.0
```

CT-H1 330.60 1.097 same as c3-h1, penalty score= 0.0
CT-N3 283.30 1.511 same as c3-n4, penalty score= 0.0
CT-HP 330.60 1.097 same as c3-hc, penalty score= 5.0
H -N3 373.20 1.030 same as hn-n4, penalty score= 0.0
N3-OS 381.80 1.421 same as n4-os, penalty score= 0.0
CT-S 215.90 1.839 same as c3-ss, penalty score= 0.0
OS-S 250.50 1.704 same as os-ss, penalty score= 0.0

ANGLE
CT-C -O 67.400 123.200 same as c3-c -o , penalty score= 0.0
CT-C -N 66.800 115.180 same as c3-c -n , penalty score= 0.0
C -CT-HC 46.900 108.770 same as c -c3-hc, penalty score= 0.0
C -N -CT 63.400 120.690 same as c -n -c3, penalty score= 0.0
C -N -H 48.300 117.550 same as c -n -hn, penalty score= 0.0
N -C -O 74.200 123.050 same as n -c -o , penalty score= 0.0
HC-CT-HC 39.400 107.580 same as hc-c3-hc, penalty score= 0.0
C -CT-N 67.000 109.060 same as c -c3-n , penalty score= 0.0
CT-CT-N 65.900 111.610 same as c3-c3-n , penalty score= 0.0
H1-CT-N 49.800 108.880 same as h1-c3-n , penalty score= 0.0
CT-N -H 45.800 117.680 same as c3-n -hn, penalty score= 0.0
CT-CT-CT 62.900 111.510 same as c3-c3-c3, penalty score= 0.0
CT-CT-HC 46.300 109.800 same as c3-c3-hc, penalty score= 0.0
C -CT-CT 63.300 111.040 same as c -c3-c3, penalty score= 0.0
C -CT-H1 47.000 108.220 same as c -c3-h1, penalty score= 0.0
CT-CT-H1 46.400 109.560 same as c3-c3-h1, penalty score= 0.0
CT-CT-N3 64.200 114.210 same as c3-c3-n4, penalty score= 0.0
CT-CT-HP 46.300 109.800 same as c3-c3-hc, penalty score= 9.5
CT-N3-H 45.900 110.110 same as c3-n4-hn, penalty score= 0.0
CT-N3-OS 67.100 107.420 same as c3-n4-os, penalty score= 0.0
HP-CT-N3 48.600 107.900 same as hc-c3-n4, penalty score= 9.5
N3-OS-S 64.800 113.340 same as na-os-ss, penalty score= 3.3
HP-CT-HP 39.400 107.580 same as hc-c3-hc, penalty score= 9.0
H -N3-OS 50.200 109.390 same as hn-n4-os, penalty score= 0.0
H -N3-H 40.600 108.300 same as hn-n4-hn, penalty score= 0.0
H1-CT-H1 39.200 108.460 same as h1-c3-h1, penalty score= 0.0
CT-CT-S 61.300 110.270 same as c3-c3-ss, penalty score= 0.0
CT-S -OS 64.600 98.210 same as c3-ss-os, penalty score= 0.0
H1-CT-S 42.100 108.760 same as h1-c3-ss, penalty score= 0.0

DIHE
CT-C -N -CT 1 0.000 0.000 -2.000 same as c3-c -n -c3
CT-C -N -CT 1 1.500 180.000 1.000 same as c3-c -n -c3, penalty score= 0.0
CT-C -N -H 4 10.000 180.000 2.000 same as X -c -n -X , penalty score= 0.0
C -CT-N -C 1 0.850 180.000 -2.000 same as c -n -c3-c
C -CT-N -C 1 0.800 0.000 1.000 same as c -n -c3-c , penalty score= 0.0
CT-CT-N -C 1 0.500 180.000 -4.000 same as c3-c3-n -c

CT-CT-N -C	1	0.150	180.000	-3.000	same as c3-c3-n -c
CT-CT-N -C	1	0.000	0.000	-2.000	same as c3-c3-n -c
CT-CT-N -C	1	0.530	0.000	1.000	same as c3-c3-n -c , penalty score= 0.0
H1-CT-N -C	6	0.000	0.000	2.000	same as X -c3-n -X , penalty score= 0.0
O -C -N -CT	4	10.000	180.000	2.000	same as X -c -n -X , penalty score= 0.0
O -C -N -H	1	2.500	180.000	-2.000	same as hn-n -c -o
O -C -N -H	1	2.000	0.000	1.000	same as hn-n -c -o , penalty score= 0.0
O -C -CT-HC	1	0.800	0.000	-1.000	same as hc-c3-c -o
O -C -CT-HC	1	0.000	0.000	-2.000	same as hc-c3-c -o
O -C -CT-HC	1	0.080	180.000	3.000	same as hc-c3-c -o , penalty score= 0.0
N -C -CT-HC	6	0.000	180.000	2.000	same as X -c -c3-X , penalty score= 0.0
O -C -CT-N	6	0.000	180.000	2.000	same as X -c -c3-X , penalty score= 0.0
N -C -CT-N	1	1.700	180.000	-1.000	same as n -c3-c -n
N -C -CT-N	1	2.000	180.000	2.000	same as n -c3-c -n , penalty score= 0.0
CT-CT-CT-N	9	1.400	0.000	3.000	same as X -c3-c3-X , penalty score= 0.0
HC-CT-CT-N	9	1.400	0.000	3.000	same as X -c3-c3-X , penalty score= 0.0
CT-CT-CT-CT	1	0.180	0.000	-3.000	same as c3-c3-c3-c3
CT-CT-CT-CT	1	0.250	180.000	-2.000	same as c3-c3-c3-c3
CT-CT-CT-CT	1	0.200	180.000	1.000	same as c3-c3-c3-c3, penalty score= 0.0
CT-CT-CT-HC	1	0.160	0.000	3.000	same as hc-c3-c3-c3, penalty score= 0.0
C -CT-CT-CT	9	1.400	0.000	3.000	same as X -c3-c3-X , penalty score= 0.0
C -CT-CT-HC	9	1.400	0.000	3.000	same as X -c3-c3-X , penalty score= 0.0
O -C -CT-CT	6	0.000	180.000	2.000	same as X -c -c3-X , penalty score= 0.0
N -C -CT-CT	1	0.100	0.000	-4.000	same as c3-c3-c -n
N -C -CT-CT	1	0.070	0.000	2.000	same as c3-c3-c -n , penalty score= 0.0
CT-CT-CT-N3	9	1.400	0.000	3.000	same as X -c3-c3-X , penalty score= 0.0
CT-CT-CT-HP	9	1.400	0.000	3.000	same as X -c3-c3-X , penalty score= 0.0
CT-CT-N3-H	9	1.400	0.000	3.000	same as X -c3-n4-X , penalty score= 0.0
CT-CT-N3-OS	9	1.400	0.000	3.000	same as X -c3-n4-X , penalty score= 0.0
CT-N3-OS-S	3	1.700	0.000	3.000	same as X -n4-os-X , penalty score= 0.0
C -CT-N -H	6	0.000	0.000	2.000	same as X -c3-n -X , penalty score= 0.0
CT-CT-N -H	6	0.000	0.000	2.000	same as X -c3-n -X , penalty score= 0.0
H1-CT-N -H	6	0.000	0.000	2.000	same as X -c3-n -X , penalty score= 0.0
O -C -CT-H1	1	0.800	0.000	-1.000	same as h1-c3-c -o
O -C -CT-H1	1	0.000	0.000	-2.000	same as h1-c3-c -o
O -C -CT-H1	1	0.080	180.000	3.000	same as h1-c3-c -o , penalty score= 0.0
N -C -CT-H1	6	0.000	180.000	2.000	same as X -c -c3-X , penalty score= 0.0
CT-CT-CT-H1	9	1.400	0.000	3.000	same as X -c3-c3-X , penalty score= 0.0
H1-CT-CT-HC	9	1.400	0.000	3.000	same as X -c3-c3-X , penalty score= 0.0
HC-CT-CT-HC	1	0.150	0.000	3.000	same as hc-c3-c3-hc, penalty score= 0.0
HC-CT-CT-N3	9	1.400	0.000	3.000	same as X -c3-c3-X , penalty score= 0.0
HC-CT-CT-HP	9	1.400	0.000	3.000	same as X -c3-c3-X , penalty score= 0.0
HP-CT-N3-H	9	1.400	0.000	3.000	same as X -c3-n4-X , penalty score= 0.0
HP-CT-N3-OS	9	1.400	0.000	3.000	same as X -c3-n4-X , penalty score= 0.0
H -N3-OS-S	3	1.700	0.000	3.000	same as X -n4-os-X , penalty score= 0.0
N -CT-CT-S	9	1.400	0.000	3.000	same as X -c3-c3-X , penalty score= 0.0

H1-CT-CT-N 9 1.400 0.000 3.000 same as X -c3-c3-X , penalty score= 0.0
CT-CT-S -OS 3 1.000 0.000 3.000 same as X -c3-ss-X , penalty score= 0.0
N3-OS-S -CT 1 2.200 0.000 2.000 same as X -os-ss-X , penalty score= 0.0
C -CT-CT-S 9 1.400 0.000 3.000 same as X -c3-c3-X , penalty score= 0.0
C -CT-CT-H1 9 1.400 0.000 3.000 same as X -c3-c3-X , penalty score= 0.0
H1-CT-CT-S 9 1.400 0.000 3.000 same as X -c3-c3-X , penalty score= 0.0
H1-CT-CT-H1 9 1.400 0.000 3.000 same as X -c3-c3-X , penalty score= 0.0
H1-CT-S -OS 3 1.000 0.000 3.000 same as X -c3-ss-X , penalty score= 0.0

IMPROPER
CT-N -C -O 1.1 180.0 2.0 Using the default value
C -CT-N -H 1.1 180.0 2.0 Same as X -X -n -hn, penalty score= 6.0 (use general term))

NONBON
CT 1.9080 0.1094 same as c3
C 1.9080 0.0860 same as c
O 1.6612 0.2100 same as o
HC 1.4870 0.0157 same as hc
N 1.8240 0.1700 same as n
N3 1.8240 0.1700 same as n4
H 0.6000 0.0157 same as hn
H1 1.3870 0.0157 same as h1
HP 0.6000 0.0157 same as hp
S 2.0000 0.2500 same as ss
OS 1.6837 0.1700 same as os

Table S4. Comparison of the equilibrium structural parameters of the S-O-NH crosslink obtained from QM/MM, CCSD/6-311G(d,p), crystallographic structures, and the derived GAFF2 force-field parameters. The percentage errors were calculated relative to the corresponding GAFF2 equilibrium parameters to assess the ability of the generated force field to reproduce the reference geometries.

Method	Method	**S-O**	**O-N**	**∢SON**	**S-O (%)**	**O-N (%)**	**∢SON (%)**
GAFF2 parameters	-S-O-NH-	1.664	1.450	112.4			
	-S-O-NH2+-	1.704	1.421	113.3			
QM/MM (EE)	-S-O-NH-	1.665	1.448	112.5	0.06	0.14	0.09
	-S-O-NH_2^+-	1.683	1.428	116.1	1.25	0.49	2.41
CCSD/6-311G(d,p)	-S-O-NH-	1.684	1.443	111.0	1.19	0.49	1.26
	-S-O-NH2+-	1.728	1.415	112.2	1.39	0.42	0.98
Experimental	PDB ID: 7ODO	1.763	1.558	107.9			
	PDB ID: 7ODP	1.769	1.543	110.6			
	PDB ID: 6ZWJ	1.770	1.457	114.2			
	PDB ID: 6ZWH	1.786	1.614	106.2			

	Experimental Average	1.772	1.543	109.7	6.095	6.03	3.3

Table S5. Sample production molecular dynamics input file used for the 200 ns simulations. All production simulations were performed in the NPT ensemble at 300 K and 1 atm using the Langevin thermostat and Monte Carlo barostat. Covalent bonds involving hydrogen atoms were constrained using the SHAKE algorithm, allowing a 2 fs integration time step. Coordinates were written in NetCDF format for subsequent trajectory analysis.

```
Production MD (200 ns)

&cntrl
 imin=0,          ! Run molecular dynamics (0 = MD, 1 = energy minimization)

 irest=1,         ! Restart simulation from a previous restart file
 ntx=5,           ! Read coordinates and velocities from the restart file

 nstlim=100000000, ! Number of MD steps (100,000,000 × 0.002 ps = 200 ns)
 dt=0.002,        ! Time step (2 fs)

 ntc=2,           ! Constrain bonds involving hydrogen atoms using SHAKE
 ntf=2,           ! Omit force evaluation for constrained bonds

 ntb=2,           ! Constant-pressure periodic boundary conditions (NPT ensemble)
 ntp=1,           ! Isotropic pressure scaling
 pres0=1.0,       ! Target pressure (1 atm)
 taup=2.0,        ! Pressure relaxation time (ps)
 barostat=2,      ! Monte Carlo barostat

 ntt=3,           ! Langevin thermostat
 gamma_ln=2.0,    ! Langevin collision frequency (ps⁻¹)
 temp0=300.0,     ! Target temperature (300 K)
 ig=-1,           ! Random seed for Langevin dynamics

 cut=10.0,        ! Non-bonded interaction cutoff (Å)

 ntpr=50000,      ! Print energies and thermodynamic information every 50,000 steps
 ntwx=50000,      ! Write trajectory coordinates every 50,000 steps
 ntwr=500000,     ! Write restart file every 500,000 steps

 ioutfm=1,        ! Write trajectory in NetCDF binary format
 iwrap=0,         ! Do not wrap atoms back into the primary periodic box
/
```
